# Methylation of elongation factor 1A by yeast Efm4 or human eEF1A-KMT2 involves a beta-hairpin recognition motif and crosstalks with phosphorylation

**DOI:** 10.1016/j.jbc.2024.105639

**Published:** 2024-01-08

**Authors:** Joshua J. Hamey, Amy Nguyen, Mahdi Haddad, Xabier Vázquez-Campos, Paige G. Pfeiffer, Marc R. Wilkins

**Affiliations:** School of Biotechnology and Biomolecular Sciences, University of New South Wales, New South Wales, Australia

**Keywords:** protein methylation, protein methyltransferase, translation elongation factor, protein cross-linking, AlphaFold, crosslinking mass spectrometry

## Abstract

Translation elongation factor 1A (eEF1A) is an essential and highly conserved protein required for protein synthesis in eukaryotes. In both *Saccharomyces cerevisiae* and human, five different methyltransferases methylate specific residues on eEF1A, making eEF1A the eukaryotic protein targeted by the highest number of dedicated methyltransferases after histone H3. eEF1A methyltransferases are highly selective enzymes, only targeting eEF1A and each targeting just one or two specific residues in eEF1A. However, the mechanism of this selectivity remains poorly understood. To reveal how *S. cerevisiae* elongation factor methyltransferase 4 (Efm4) specifically methylates eEF1A at K316, we have used AlphaFold-Multimer modeling in combination with crosslinking mass spectrometry (XL-MS) and enzyme mutagenesis. We find that a unique beta-hairpin motif, which extends out from the core methyltransferase fold, is important for the methylation of eEF1A K316 *in vitro*. An alanine mutation of a single residue on this beta-hairpin, F212, significantly reduces Efm4 activity *in vitro* and in yeast cells. We show that the equivalent residue in human eEF1A-KMT2 (METTL10), F220, is also important for its activity towards eEF1A *in vitro*. We further show that the eEF1A guanine nucleotide exchange factor, eEF1Bα, inhibits Efm4 methylation of eEF1A *in vitro*, likely due to competitive binding. Lastly, we find that phosphorylation of eEF1A at S314 negatively crosstalks with Efm4-mediated methylation of K316. Our findings demonstrate how protein methyltransferases can be highly selective towards a single residue on a single protein in the cell.

Protein methylation is one of the most important post-translational modifications in the eukaryotic cell. Alongside the well-understood role of histone methylation in transcriptional regulation, non-histone methylation is now known to be central to the regulation of processes including cellular signaling, RNA splicing, protein synthesis, metabolism and cellular respiration (1, 2, 3, 4, 5). This can occur through mechanisms such as regulation of protein activity, localization, protein-protein interactions or crosstalk with other post-translational modifications (3). Methylation predominantly occurs on lysine and arginine residues, although it also occurs on other residues such as histidine, glutamine and at the N- and C-termini of proteins (4, 6, 7).

Protein lysine methylation is catalyzed by *S*-adenosyl-L-methionine (AdoMet)-dependent methyltransferases, which can transfer up to three methyl groups to the ε-amino of lysine sidechains. These enzymes largely belong to one of two families: the SET domain methyltransferase family (8) and the seven-beta-strand (7βS) methyltransferase family (9, 10). SET domain enzymes exclusively methylate proteins, while 7βS methyltransferases can methylate proteins, nucleic acids, lipids and metabolites (11). While the mechanisms underpinning substrate selectively of SET domain enzymes have been widely studied, substrate selectivity for 7βS enzymes is less well understood. SET domain enzymes tend to recognize their substrates as linear amino acid sequences, and thus studies of their substrate selectivity are amenable to peptide-based approaches (12, 13). In contrast, 7βS enzymes tend to recognize the three-dimensional structural features of their substrates, making investigations into the mechanisms unpinning their substrate selection more difficult. In fact, only a handful of structures of 7βS methyltransferases bound to whole-protein substrates have been determined to-date: bacterial PrmA with ribosomal protein L11 (14), human DOT1L with a nucleosome substrate (15), and recently, human VCP-KMT with VCP/p97 (16, 17). Additionally, a model of human eEF2-KMT/FAM86A bound to eEF2 has recently been determined using AlphaFold (18). All these structures show that contacts distal to the methyltransferase active site are critical for binding and methylation. Additionally, most 7βS enzymes appear to be incredibly specific, with many known to methylate just a single amino acid in a single protein (10). Interestingly, these enzymes also tend to be small, with the core methyltransferase fold making up the majority of their sequence in many cases (10, 19). When these proteins do have additional domains, they appear to be essential for their enzymatic activity (18, 19). It therefore remains unclear how the vast majority of 7βS methyltransferases recognize and methylate their substrate proteins.

Eukaryotic translation elongation factor 1A (eEF1A) is an abundant and highly conserved protein essential for eukaryotic life. Its canonical role is to facilitate the accommodation of aminoacyl-tRNA (aa-tRNA) in the A-site of the ribosome during translation elongation (20). It also has several other cellular functions, including in actin polymerization, proteasomal protein degradation, nuclear export and the heat shock response (21). eEF1A is made up of three domains. Domain 1 (also called the G domain) is its GTPase domain which hydrolyzes GTP during aa-tRNA accommodation in the ribosome (22). Domains 2 and 3 are additionally involved in binding aa-tRNA and the guanine nucleotide exchange factor, protein eEF1Bα, and may be involved in non-canonical roles such as actin binding (22). eEF1A exists in multiple different conformations depending on the orientation of domain 1 with respect to domains 2 and 3, which form a single structural unit (23, 24). Co-crystal structures of the eEF1A:eEF1Bα complex have shown that eEF1Bα binds across domains 1 and 2 of eEF1A, inducing a partially closed conformation of eEF1A (25). Structures of the ternary eEF1A:aa-tRNA:GTP complex bound to the ribosome show that eEF1A adopts a completely closed and compact conformation, with domain 1 making extensive contacts with domain 3 (26, 27). eEF1A:GDP, on the other hand, adopts a completely open, extended conformation (24), which has also recently been described to occur when eEF1A is bound to the ribosome during aa-tRNA accommodation (28).

eEF1A is subject to many different types of post-translational modifications (PTMs), including methylation (29, 30), phosphorylation (31), ubiquitination (32), acetylation (33), and the unique modifications glycerylphosphorylethanolamine (34) and glutaminylation (35). Many of these modifications are conserved across eukaryotes. Most prominently, eEF1A is highly methylated, being the protein targeted by the largest number of dedicated methyltransferases in the eukaryotic cell besides histone H3 (29, 30). There are five eEF1A-specific protein methyltransferases in both *Saccharomyces cerevisiae* and human, each of which targets distinct residues (29, 30). Two eEF1A methylation sites, at K79 and K316 (yeast numbering), are found across eukaryotes (29). In *S. cerevisiae* and human, these are catalyzed by orthologous enzymes: yeast Efm5 and human eEF1A-KMT2 trimethylate eEF1A at K79 (36, 37), while yeast Efm4 dimethylates eEF1A at K316 and its human ortholog, eEF1A-KMT2, trimethylates eEF1A at the equivalent residue, K318 (38, 39). While the function of these conserved methylation events remains largely unknown, K316 is positioned at the interface with the ribosome, suggesting a role in translation (29). eEF1A is also phosphorylated at over a dozen sites, most of which are conserved between yeast and human (31). eEF1A phosphorylation has been reported to regulate protein synthesis and actin dynamics, and to affect eEF1A activity, stability and protein interactions (31, 40, 41, 42, 43). Despite being extensively post-translationally modified, it is not yet known whether any eEF1A PTMs engage in crosstalk with each other, whereby PTMs positively or negatively affect the deposition of other PTMs.

Here we combine AlphaFold modeling, crosslinking mass spectrometry and enzyme mutagenesis to reveal the basis of specificity for a highly conserved eEF1A methyltransferase, known as Efm4 in *S. cerevisiae* and eEF1A-KMT2 (METTL10) in human. We find that a short beta-hairpin motif in Efm4/eEF1A-KMT2 binds eEF1A in a conserved hydrophobic pocket, and that mutation of a single conserved phenylalanine in this beta-hairpin dramatically reduces Efm4 and eEF1A-KMT2 methylation of eEF1A *in vitro*. Furthermore, mutation of this phenylalanine in yeast cells abolishes eEF1A K316 methylation *in vivo*. Interestingly, this eEF1A hydrophobic pocket is also bound by eEF1Bα, and we show that eEF1Bα inhibits Efm4 methylation of K316 *in vitro*. We further show that phosphorylation of a nearby serine (S314) partially inhibits eEF1A K316 methylation by Efm4, providing the first known instance of PTM crosstalk on eEF1A.

## Results

### AlphaFold-multimer modeling and crosslinking mass spectrometry validation of the Efm4:eEF1A complex

**Figure 1:**
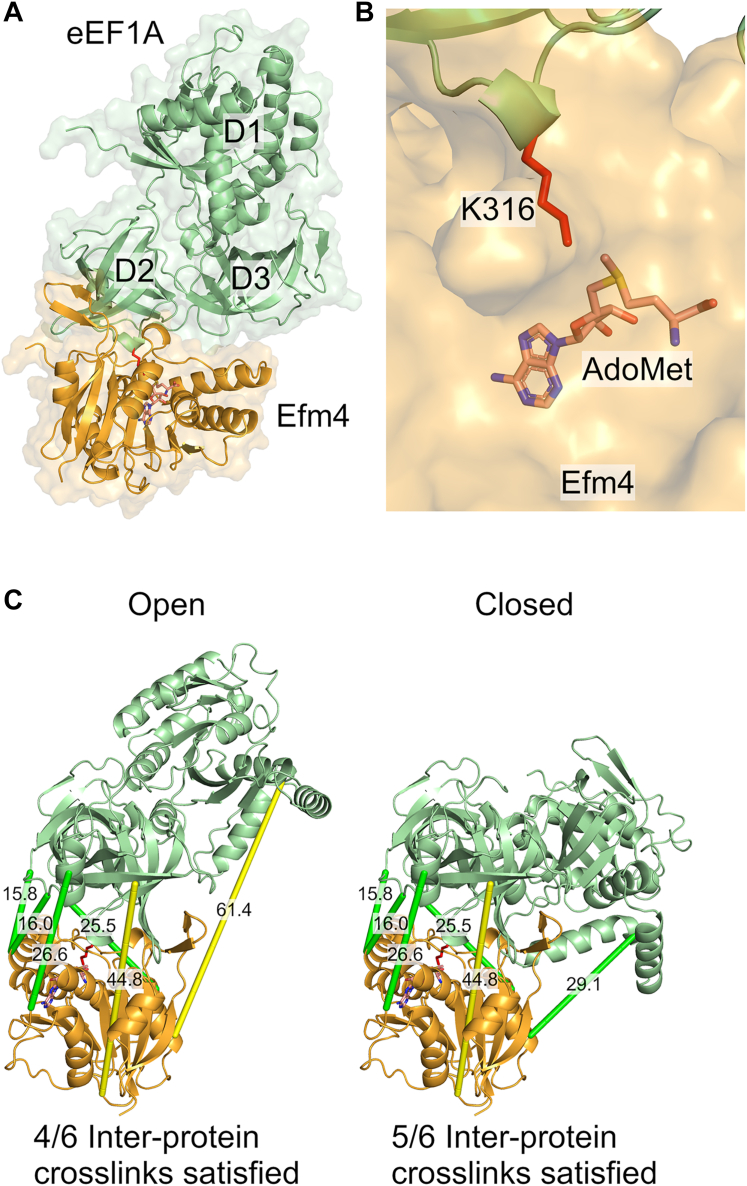
**AlphaFold-multimer model of Efm4 bound to eEF1A and validation by crosslinking mass spectrometry.***A*, *top*-ranked AlphaFold-Multimer model of Efm4 (*orange*) bound to eEF1A (*green*) with AdoMet (shown as *sticks*) docked into Efm4. The three domains of eEF1A are labeled as D1, D2, and D3 respectively. *B*, model in (*A*) showing proximity of eEF1A K316 (*red* sticks) to the transferred methyl group of AdoMet (*sticks*) in Efm4 (*orange* surface). *C*, crosslinking mass spectrometry (XL-MS) analysis of the Efm4:eEF1A complex was carried out with DSSO. Purified Efm4 was incubated with eEF1A purified from a ΔEFM4 yeast strain in the presence of DSSO and AdoMet. Proteins were then digested with trypsin, GluC, or chymotrypsin, and the resulting crosslinked peptides were detected by LC-MS/MS. The six detected unique crosslinks were mapped into the AlphaFold-Multimer model (rank 1) of Efm4:eEF1A in the “open” conformation (*left*) and the “closed” conformation (*right*). The “closed” conformation of eEF1A was generated by aligning domain 1 of eEF1A from the AlphaFold-Multimer model to domain 1 of eEF1A bound to the ribosome (PDB ID: 4CXG). In the “closed” conformation, five crosslinks were within the expected 30 Å distance for DSSO (shown in *green*), while one was >30 Å (shown in *yellow*).

*S. cerevisiae* elongation factor methyltransferase 4 (Efm4) is a highly conserved 7βS methyltransferase which specifically methylates translation elongation factor 1A (eEF1A) at K316. To gain insight into the mechanisms underpinning this substrate specificity, we generated AlphaFold-Multimer models of Efm4 bound to eEF1A (Figs. 1*A* and S1). All five models had high interface predicted TM (ipTM) scores (0.835–0.903), high average pLDDTs (86.3–87) and were in good agreement with each other (Fig. S1). We therefore carried out further analyses with reference to the rank 1 model (highest ipTM score). Efm4 was modeled with the expected 7βS fold disrupted only by a short beta-hairpin motif extending out from the core fold (Fig. S2*A*). The predicted structure of eEF1A was largely identical to that seen in crystal structures, only differing by the degree to which domain 1 is ‘closed’ with respect to domains 2 and 3, and with K316, which is located on domain 2, in a near-identical position (Fig. S2*B*). In the model, Efm4 was predicted to interact with domains 2 and 3 of eEF1A, and most strikingly, eEF1A K316 was predicted to be bound at the active site of Efm4, proximal to the transferred methyl group of the methyl donor *S*-adenosyl-L-methionine (AdoMet) (Figs. 1*B* and S1). It is important to note that the EFM4 gene begins 84 nucleotides downstream of the currently annotated start site (*i.e.* EFM4 genomic co-ordinates are chrIX:242,027 – 242,716 rather than chrIX:241,943 – 242,716), at the methionine currently annotated in position 29, as supported by mRNA sequencing and ribosome footprint sequencing data (Fig. S3). Furthermore, deletion of this upstream region from the Efm4 protein does not affect its activity *in vitro* (Fig. S4). We therefore carried out all subsequent analyses on a form of Efm4 without this upstream region and have numbered all residues accordingly.

To validate the predicted structure of the Efm4:eEF1A complex, we carried out chemical crosslinking followed by mass spectrometry (XL-MS). Efm4 was expressed and purified from *E. coli* while eEF1A was purified from a ΔEFM4 yeast strain by the use of a chromosomally-integrated hexahistidine tag (see Methods) to generate eEF1A which should lack K316 methylation. Purified Efm4 and eEF1A were then crosslinked with DSSO in the presence of the co-factor AdoMet, before digestion with either trypsin, GluC or chymotrypsin and analysis of crosslinked peptides by LC-MS/MS. We detected methylation of eEF1A K316 (Fig. S5), indicating that Efm4 and eEF1A interacted during the crosslinking reaction. Six unique inter-protein crosslinks between Efm4 and eEF1A were identified (Fig. S6), four of which were within the expected maximum Cα-Cα distance for DSSO of 30 Å when mapped onto the highest ranked Efm4:eEF1A model (Fig. 1*C*, left). A fifth crosslink between eEF1A K55 and Efm4 K203 could be explained by the conformational flexibility of eEF1A domain 1, as the distance between these two residues is <30 Å when eEF1A is in its “closed” confirmation (Fig. 1*C*, right). Given that Efm4 necessarily binds eEF1A such that K316 is positioned in its active site, these crosslinks support the AlphaFold-Multimer model by confirming the orientation and rotation of Efm4 relative to eEF1A. Overall, our AlphaFold-Multimer model of Efm4 bound to eEF1A demonstrates the expected binding to the target residue and is in agreement with our XL-MS data.

### Efm4 residues predicted to bind eEF1A are critical for its activity towards K316 *in vitro* and *in vivo*

**Figure 2:**
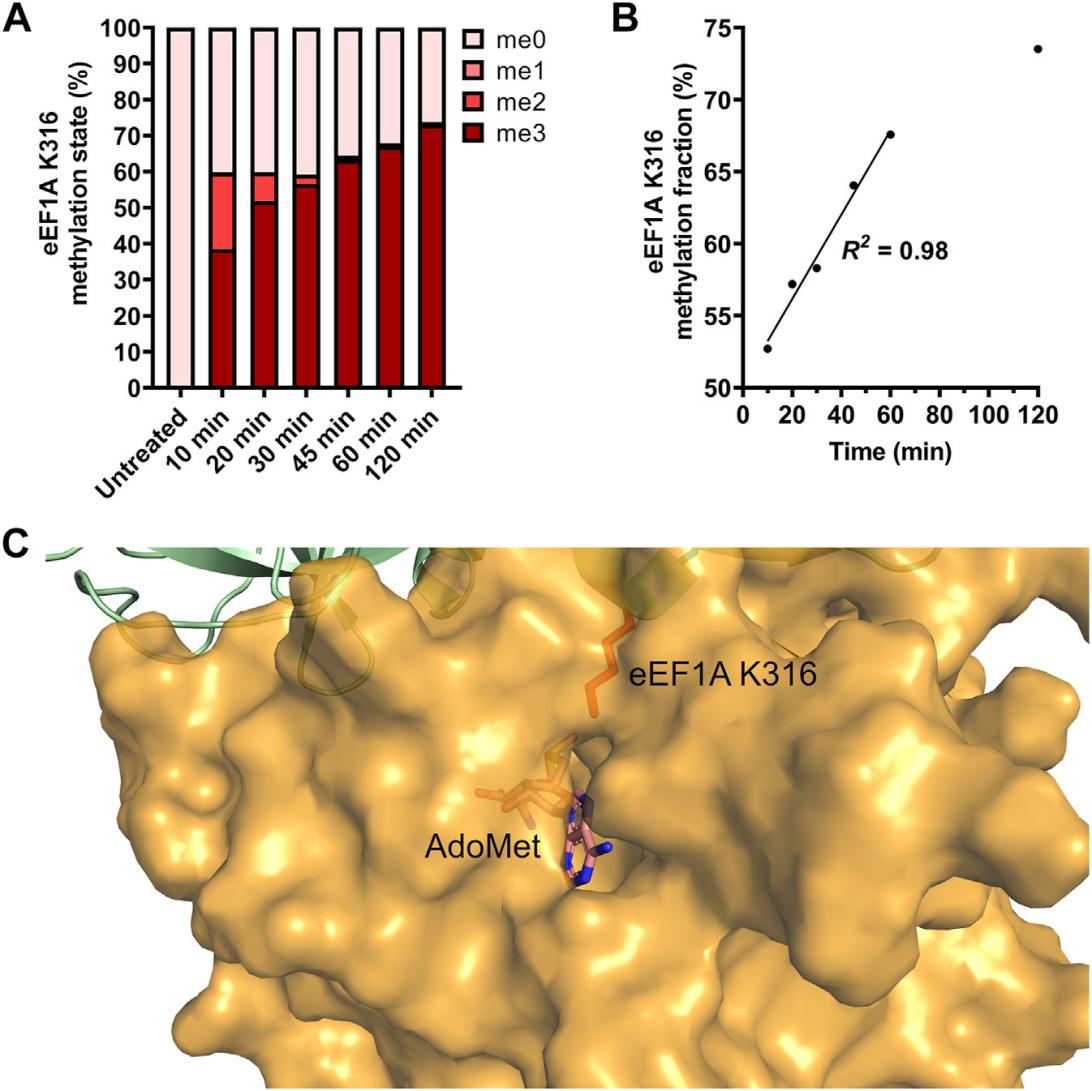
**Efm4 catalyzes eEF1A K316 dimethylation processively.***A* and *B*, Efm4 time-series methylation assay. Purified WT Efm4 (3 μM) was incubated with eEF1A (from ΔEFM4) (2 μM) in the presence of AdoMet at 30 °C, for the indicated times. Proteins were separated by SDS-PAGE (see Fig. S7), eEF1A gel bands digested with AspN, and the resulting eEF1A K316 methylation was detected by LC-MS/MS and quantification of AspN-generated peptide DNVGFNVKNVSVK (K316 underlined) in its triply-charged state. *A*, relative levels of eEF1A K316 methylation states. *B*, eEF1A K316 methylation fraction relative to 100% trimethylated K316, with a line of best fit shown for 10–60 min. *C*, AlphaFold-Multimer model of Efm4:eEF1A (rank 1) showing a channel in Efm4 (shown as an *orange* surface structure) through which AdoMet (shown as *sticks*) could be exchanged, while Efm4 remains bound to eEF1A K316 (shown as *red sticks*).

Our AlphaFold-Multimer model revealed Efm4 residues which likely interact with eEF1A to facilitate K316 methylation. To investigate these further, we first carried out *in vitro* assays to probe Efm4 methylation of eEF1A. We again used Efm4 expressed and purified from *E. coli* and eEF1A purified from a ΔEFM4 yeast strain. Purified Efm4 and eEF1A were incubated in the presence of AdoMet, in a time series manner (Fig. S7), and analyzed by LC-MS/MS. In the absence of Efm4, eEF1A K316 was found to be completely unmethylated (Fig. 2*A*). The time series assay revealed that Efm4 rapidly catalyzes dimethylation of K316 (<10 min), while subsequent trimethylation proceeds more slowly (Fig. 2*A*). This is in agreement with the fact that dimethylation is predominantly observed *in vivo* (44, 45). Importantly, Efm4 methylation activity was linear for at least the first hour under the conditions and concentrations used (Fig. 2*B*). The fact that a substantial amount of K316 remains unmethylated while dimethylation and trimethylation occur suggests a processive mechanism of methylation, whereby Efm4 binds eEF1A and catalyzes multiple rounds of methylation before dissociating. In agreement with this, the predicted structure of Efm4:eEF1A shows a channel through which *S*-adenosyl-L-homocysteine (AdoHcy) might freely exchange for AdoMet while Efm4 remains bound to eEF1A (Fig. 2*C*).

**Figure 3:**
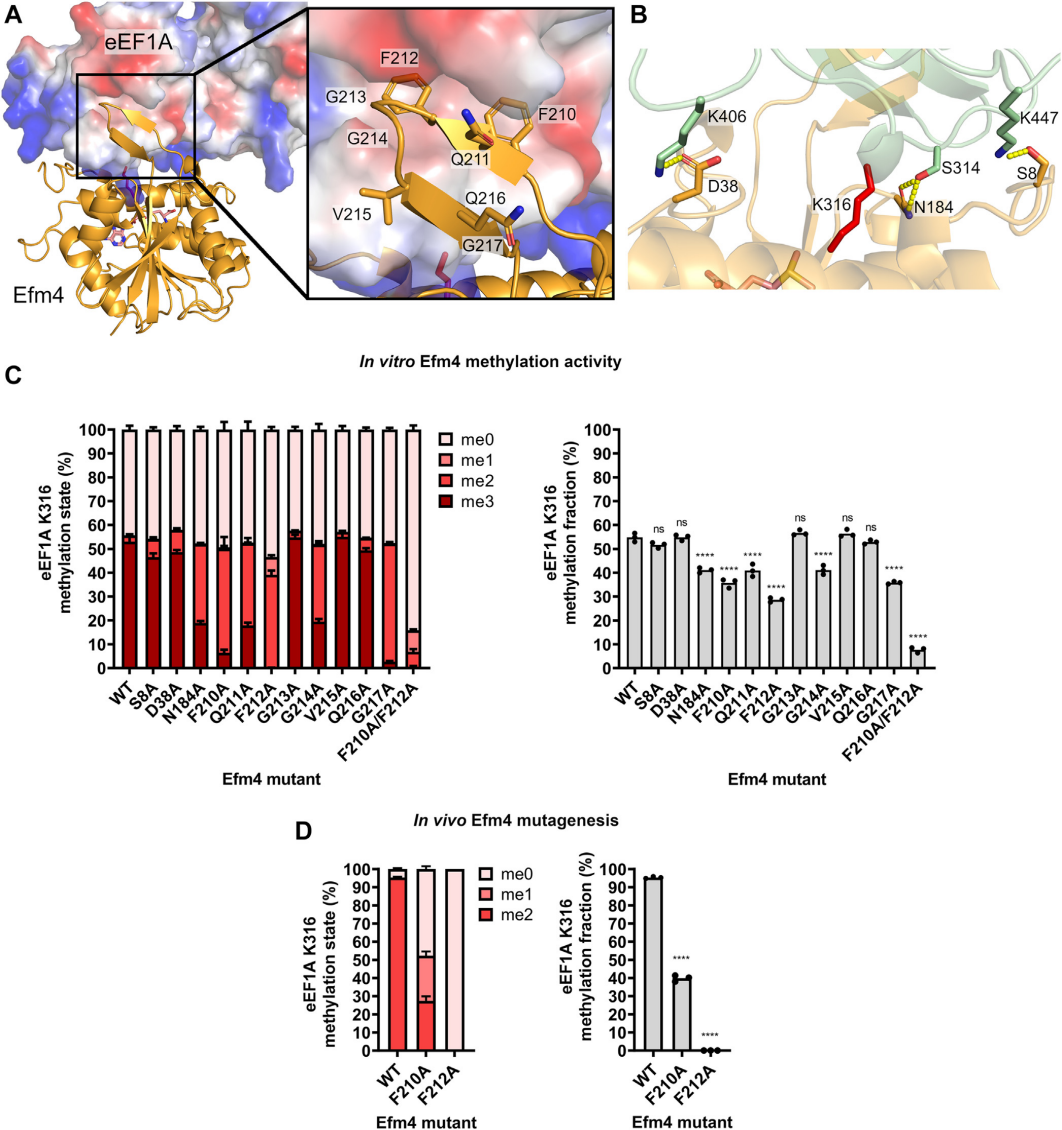
**A beta-hairpin on Efm4 is critical for its activity towards eEF1A K316 *in vitro* and *in vivo*.***A*, AlphaFold-Multimer model of Efm4:eEF1A (rank 1) showing a beta-hairpin extending from Efm4 binding a hydrophobic pocket in domain 2 of eEF1A. Efm4 (*orange*) is shown as a cartoon structure. eEF1A is shown as its surface electrostatic potential (*blue* = positive, *red* = negative, *white* = neutral). Inset: sidechains of residues on Efm4 beta-hairpin are shown as *sticks* (nitrogen = *blue*, oxygen = *red*). *B*, model in (*A*) showing predicted polar contacts between Efm4 (*orange*) and eEF1A (*green*). K316 is show as *red* sticks. Hydrogen bonds are shown as *yellow* dashes. *C*, *in vitro* methylation assays of Efm4 mutants. Purified WT or mutant Efm4 (3 μM) were incubated with eEF1A (from ΔEFM4) (2 μM) in the presence of AdoMet for 30 min at 30 °C. Assays were carried out in triplicate. Proteins were separated by SDS-PAGE (see Fig. S8), eEF1A gel bands digested by AspN, and the resulting eEF1A K316 methylation was detected by LC-MS/MS and quantification of AspN-generated peptide DNVGFNVKNVSVK (K316 underlined) in its triply-charged state. *Left*: Relative levels of eEF1A K316 methylation states. Error bars show one SD. *Right*: eEF1A K316 methylation fraction relative to 100% trimethylated K316. Methylation fractions from mutant Efm4 were compared to WT Efm4 using an ordinary one-way ANOVA with a post hoc Dunnett’s multiple comparisons test (ns: not significant, ∗∗∗∗*p* ≤ 0.0001). *D*, F210A or F212A mutation of Efm4 reduces or ablates eEF1A K316 methylation *in vivo*. All three clones of WT, F210A, and F212A Efm4 genomic mutants (see Table 1) were analyzed for their levels of eEF1A K316 methylation by parallel reaction monitoring of GluC peptide QGVPGDNVGFNVKNVSVKE (K316 underlined). *Left*: Relative levels of eEF1A K316 methylation states. *Right*: eEF1A K316 methylation fraction relative to 100% dimethylated K316. Methylation fractions from mutant Efm4 were compared to WT Efm4 using an ordinary one-way ANOVA with a post hoc Dunnett’s multiple comparisons test (∗∗∗∗: *p* ≤ 0.0001).

The predicted interface of Efm4 and eEF1A includes a prominent, nine-residue beta-hairpin motif inserted between β6 and β7 in the core 7βS fold of Efm4, which putatively binds to a hydrophobic pocket in domain 2 of eEF1A (Fig. 3*A*). There are also several other residues on the core methyltransferase domain which appear to form polar contacts with eEF1A residues on domains 2 and 3 (Fig. 3*B*). To determine whether these Efm4 residues are of particular importance for eEF1A methylation, we generated Efm4 carrying alanine mutations at each of these residues and carried out *in vitro* methylation assays (Fig. S8). Strikingly, mutations of five of the nine residues in the beta-hairpin (F210, Q211, F212, G214, and G217) showed a significant reduction in eEF1A methylation, mostly due to a severe reduction in trimethylation, indicating that these amino acids are required for full Efm4 activity (Fig. 3*C*). Efm4 F210 and F212, which appear to bind the eEF1A hydrophobic pocket most directly, are particularly important for Efm4 methyltransferase activity. Expectedly, a F210A/F212A double mutant catalyzed less than 20% of the methylation made by WT Efm4, which was a result of a substantial reduction in dimethylation and a near complete lack of trimethylation being catalyzed by this mutant (Fig. 3*C*). In contrast, the three alanine mutations on amino acids in the core domain of Efm4 led to a reduction in Efm4 activity in only one case (N184A) (Fig. 3*C*).

Efm4 F210 and F212 appear to be most buried within the eEF1A domain 2 hydrophobic pocket (Fig. 3*A*), explaining their importance for Efm4 activity. F212, in particular, appears to bind deep in the pocket formed by several eEF1A residues (I254, I257, V260, S289, E291, G307, F308, and N309). To further confirm the importance of F210 and F212 for Efm4 methylation of eEF1A, we tested the effect of F210A and F212A mutations on eEF1A K316 methylation *in vivo* by introducing these mutations separately into the chromosomal copy of EFM4. Three clones of each mutant were generated, as well as three clones of the WT EFM4 control strain which contains the URA3 marker used for selection as well as a C-terminal hexahistidine tag (see Table 1). We confirmed that the F210A and F212A mutants expressed similar to WT Efm4 by enriching them *via* their hexahistidine tags and analyzing their levels by LC-MS/MS (Fig. S9). Parallel reaction monitoring of a GluC peptide containing eEF1A K316 revealed the expected near-stoichiometric levels of dimethylation in the WT EFM4 control strain (Fig. 3*D*). In agreement with the *in vitro* data, the F210A mutant strain had significantly less K316 methylation than WT, with a substantial portion of K316 being un- and mono-methylated (Fig. 3*D*). Remarkably, the F212A mutant strain showed a complete loss of K316 methylation (Fig. 3*D*). This indicates that F212 is essential for Efm4 *in vivo* activity. Overall, our data show that residues in a beta-hairpin on Efm4, particularly F210 and F212, are critical for its methylation of eEF1A K316.

**Table 1:** *Saccharomyces cerevisiae* strains used in this study

Strain	Genotype	Description	Purpose	Direct background strain	Source
BY4741	MATa his3Δ1 leu2Δ0 met15Δ0 ura3Δ0	Wild-type	Background strain	-	Euroscarf
ΔEFM4	MATa his3Δ1 leu2Δ0 met15Δ0 ura3Δ0 EFM4::kanMX4	EFM4 knockout	Background strain	BY4741	Euroscarf
EFM4-His	EFM4::EFM4- 6xHis (URA3)	-WT EFM4 with C-terminal 6xHis-tag; downstream URA3	Control for EFM4 mutant strains	BY4741	This study
EFM4-F210A-His	EFM4::EFM4-F210A-6xHis (URA3)	-EFM4 with F210A mutation and C-terminal 6xHis-tag; downstream URA3	Efm4 *in vivo* mutagenesis	BY4741	This study
EFM4-F212A-His	EFM4::EFM4-F212A-6xHis (URA3)	-EFM4 with F212A mutation and C-terminal 6xHis-tag; downstream URA3	Efm4 *in vivo* mutagenesis	BY4741	This study
ΔEFM4 pD1204-eEF1A1	EFM4::kanMX4, pD1204-eEF1A1	-EFM4 knockout -Human eEF1A1 under galactose-inducible promoter	Purification of WT eEF1A1 for eEF1A-KMT2 methylation assays	ΔEFM4	This study
ΔEFM4 pD1204-eEF1A2	EFM4::kanMX4, pD1204-eEF1A2	-EFM4 knockout -Human eEF1A2 under galactose-inducible promoter	Purification of WT eEF1A2 for eEF1A-KMT2 methylation assays	ΔEFM4	This study
TEF1-His	TEF1::TEF1-6xHis (URA3)	-WT TEF1 with C-terminal 6xHis-tag; downstream URA3	Control for eEF1A mutant strains	BY4741	This study
TEF1-His ΔTEF2	TEF1::TEF1-6xHis (URA3), TEF2::natNT2	-TEF1 with C-terminal 6xHis-tag; downstream URA3 -TEF2 knockout	Purification of eEF1A for phospho-enrichment	TEF1-His	This study
TEF1-S314A-His	TEF1::TEF1-S314A-6xHis (URA3)	-TEF1 with S314A mutation and C-terminal 6xHis-tag; downstream URA3	eEF1A *in vivo* mutagenesis	BY4741	This study
TEF1-S314D-His	TEF1::TEF1-S314D-6xHis (URA3)	-TEF1 with S314D mutation and C-terminal 6xHis-tag; downstream URA3	eEF1A *in vivo* mutagenesis	BY4741	This study
ΔEFM4 TEF1-His	EFM4::kanMX4, TEF1::TEF1-6xHis (URA3)	-EFM4 knockout -WT TEF1 with C-terminal 6xHis-tag; downstream URA3	Purification of WT eEF1A for Efm4 methylation assays	ΔEFM4	This study
ΔEFM4 TEF1-S314A-His	EFM4::kanMX4, TEF1::TEF1-S314A-6xHis (URA3)	-EFM4 knockout -TEF1 with S314A mutation and C-terminal 6xHis-tag, downstream URA3	Purification of S314A eEF1A for Efm4 methylation assays	ΔEFM4	This study
ΔEFM4 TEF1-S314D-His	EFM4::kanMX4, TEF1::TEF1-S314D-6xHis (URA3)	-EFM4 knockout -TEF1 with S314D mutation and C-terminal 6xHis-tag, downstream URA3	Purification of S314D eEF1A for Efm4 methylation assays	ΔEFM4	This study

### eEF1Bα inhibits Efm4 methylation of eEF1A

**Figure 4:**
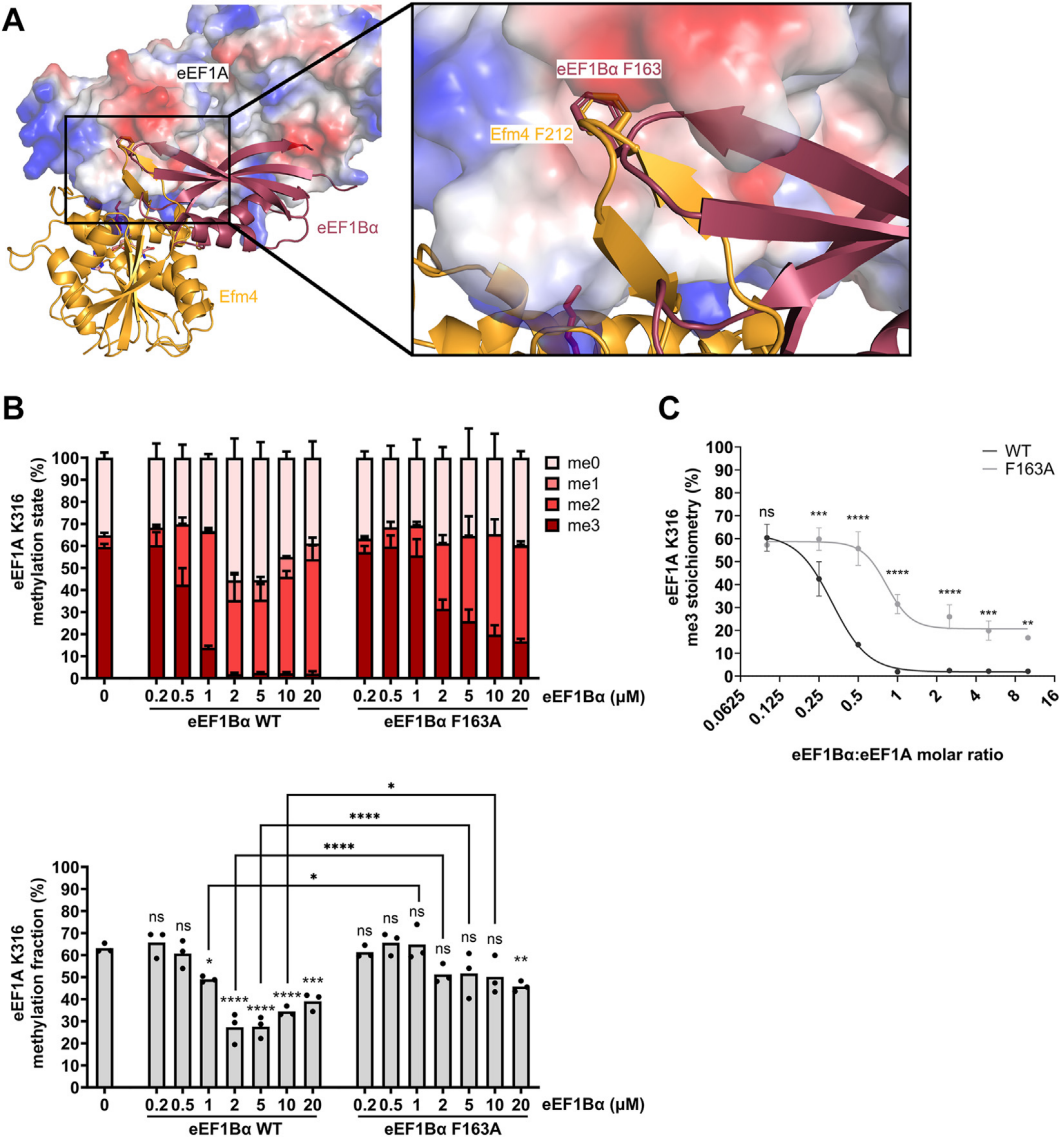
**eEF1Bα inhibits Efm4 methylation of eEF1A K316 *in vitro*.***A*, the co-crystal structure of eEF1A and eEF1Bα (PDB ID: 1F60) was aligned to the AlphaFold-Multimer model of Efm4:eEF1A (rank 1), by alignment of domains 2 and 3 of eEF1A. Efm4 (*orange*) and eEF1Bα (*dark red*) are shown as cartoon structures. eEF1A is shown as its surface electrostatic potential (*blue* = positive, *red* = negative, *white* = neutral). *B* and *C*, *in vitro* methylation assays of Efm4 in the presence of eEF1Bα. Purified WT Efm4 (3 μM) was incubated with eEF1A (from ΔEFM4) (2 μM) in the presence of varying concentrations of eEF1Bα WT or F163A and with AdoMet for 30 min at 30 °C. Proteins were separated by SDS-PAGE (see Fig. S10), eEF1A gel bands digested by AspN, and the resulting eEF1A K316 methylation was detected by LC-MS/MS and quantification of AspN-generated peptide DNVGFNVKNVSVK (K316 underlined) in its triply-charged state. *B*, *Top*: Relative levels of eEF1A K316 methylation states. *Bottom*: eEF1A K316 methylation fraction relative to 100% trimethylated K316. Methylation fractions from assays in the presence of eEF1Bα (WT and F163A) were compared to the assay without eEF1Bα (0 μM) using an ordinary one-way ANOVA with a post hoc Dunnett’s multiple comparisons test. Methylation fractions from assays in the presence of the same concentrations of WT or F163A mutant eEF1Bα were compared using an ordinary one-way ANOVA with a post hoc Šídák’s multiple comparisons test (nonsignificant results for 0.2 μM, 0.5 μM, and 20 μM not shown). ns: not significant, ∗*p* ≤ 0.05, ∗∗*p* ≤ 0.01, ∗∗∗*p* ≤ 0.001, ∗∗∗∗*p* ≤ 0.0001. *C*, relative levels of eEF1A K316 trimethylation are significantly higher for Efm4 methylation assays coincubated with F163A mutant eEF1Bα compared with WT eEF1Bα, at eEF1Bα:eEF1A molar ratios of 0.25 and higher. Line of best fit: four-parameter dose-response curve; WT r^2^ = 0.98, F163A r^2^ = 0.93.

eEF1Bα is the nucleotide exchange factor for eEF1A. Co-crystal structures have shown that eEF1Bα partially binds eEF1A through interacting with the hydrophobic pocket on eEF1A domain 2 (25). eEF1Bα F163 binds this pocket, and an F163A mutant has an approximately 6.5-fold increase in K_d_ relative to WT (46). Structural alignment of our Efm4:eEF1A AlphaFold-Multimer model with an eEF1A-eEF1Bα crystal structure shows that Efm4 F212 and eEF1Bα F163 bind the hydrophobic pocket in the same way (Fig. 4*A*). This suggests that eEF1Bα may inhibit Efm4 binding to eEF1A and therefore its methylation at K316. It also suggests that a F163A mutation of eEF1Bα may reduce this inhibition. To test this, we expressed and purified WT and F163A mutant eEF1Bα from *E. coli* and titrated them into an Efm4 methylation assay of eEF1A (Fig. S10). The resulting levels of K316 methylation were then detected by LC-MS/MS. While low concentrations of WT eEF1Bα had no effect on Efm4 activity, WT eEF1Bα concentrations over 50% of the 2 μM eEF1A concentration (*i.e.* greater than 1 μM) significantly reduced eEF1A K316 methylation by Efm4 (Fig. 4*B*). For F163A mutant eEF1Bα, however, only at a 10× molar excess of eEF1Bα relative to eEF1A (*i.e.* at 20 μM eEF1Bα F163A) did we observe a significant reduction in Efm4 activity (Fig. 4*B*). Furthermore, co-incubation of F163A mutant eEF1Bα with Efm4 and eEF1A led to significantly higher levels of K316 methylation, compared with WT eEF1Bα, at eEF1Bα concentrations of 1 μM, 2 μM, 5 μM and 10 μM (Fig. 4*B*). The effect was most prominent on the levels of K316 trimethylation, which were significantly different between WT and F163A mutant eEF1Bα at a molar ratio to eEF1A of 0.25 or higher (*i.e.* 0.5 μM eEF1Bα and higher) (Fig. 4*C*). This indicates that the F163A mutation of eEF1Bα significantly reduces its inhibitory effect on Efm4 methylation of eEF1A K316. Overall, eEF1Bα is able to inhibit Efm4 methylation of eEF1A and a F163A mutation of eEF1Bα reduces this inhibition, consistent with the prediction that they competitively bind the hydrophobic pocket in eEF1A domain 2.

### A non-canonical isoform of eEF1A-KMT2 is the human homolog of Efm4

Efm4 is a highly conserved enzyme in eukaryotes. In human cells, knockdown of Efm4 homolog eEF1A-KMT2 (also known as METTL10) reduces methylation of eEF1A K318 (equivalent to yeast K316), and the mouse homolog can catalyze K318 methylation *in vitro* (39). We therefore sought to investigate whether human eEF1A-KMT2 interacts with eEF1A through similar mechanisms as Efm4. However, our attempts to generate AlphaFold-Multimer models of human eEF1A-KMT2 bound to eEF1A1 or eEF1A2 (the two human eEF1A paralogs) using the canonical isoform of eEF1A-KMT2 (eEF1A-KMT2-201; UniProt: Q5JPI9; ENSEMBL: ENST00000368836.7; RefSeq: NP_997719.2) were not successful. These models were of poor quality (ipTM scores 0.169–0.2) and eEF1A K318 was not bound at the active site of eEF1A-KMT2 (Figs. S11 and S12). In contrast, models generated with a secondary eEF1A-KMT2 isoform (eEF1A-KMT2-207; UniProt: A0A494BZY7; EMSEMBL: ENST00000652548.1; RefSeq: NP_001403172.1) produced higher quality predictions (ipTM scores 0.556–0.838) wherein K318 of both eEF1A1 and eEF1A2 are bound at its active site (Figs. S13 and S14). Given these differing results, we therefore investigated the expression of these two eEF1A-KMT2 isoforms in human cells.

**Figure 5:**
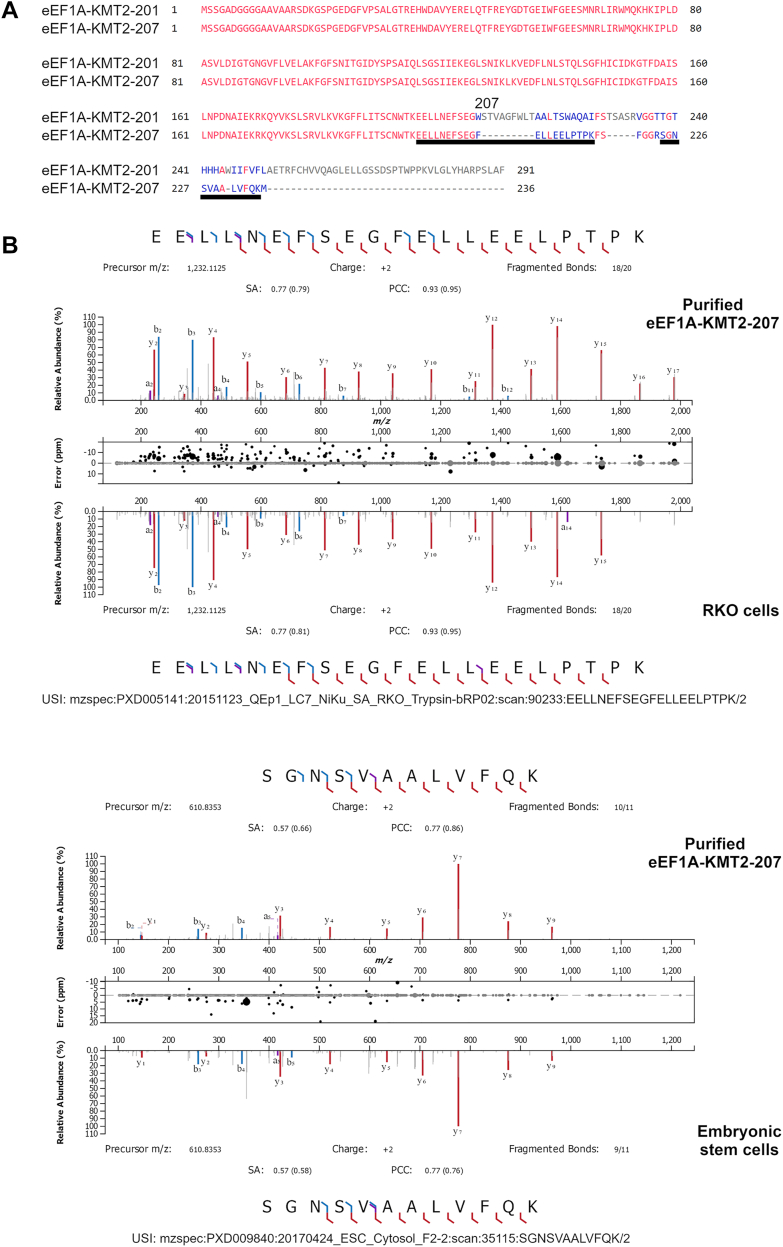
**A noncanonical isoform of eEF1A-KMT2 is the predominantly expressed protein.***A*, alignment of the canonical isoform of eEF1A-KMT2 (eEF1A-KMT2-201) and a secondary isoform (eEF1A-KMT2-207), showing that they differ only at their C-terminal regions from residue 207 onwards. Identical residues are *red*, different residues are *blue*, and gaps are *gray*. The two underlined regions of eEF1A-KMT2-207 indicate the peptides confirmed in (*B*) and Fig. S16. Alignment was generated in COBALT (NCBI). *B*, comparison of MS/MS spectra generated from purified eEF1A-KMT2-207 and from public proteomic datasets, confirming the identity of two eEF1A-KMT2-207-specific peptides. These peptides were also confirmed in two other cell lines (see Fig. S16). Spectra were graphed and compared using the Universal Spectrum Explorer (91), with the following settings: fragment ions: a, b, y; fragment ion charge states: 1+, 2+; fragment annotation tolerance: 20 ppm; annotation intensity threshold: 5% base peak.

eEF1A-KMT2-201 and eEF1A-KMT2-207 are the only eEF1A-KMT2 isoforms predicted to encode full, stable proteins. They are identical except in their C-terminal regions, from position 207 onwards, due to differential inclusion of exon 6 (Fig. 5*A*). eEF1A-KMT2-201 includes exon 6 and terminates at a stop codon on the boundary between exons 6 and 7. eEF1A-KMT2-207, on the other hand, excludes exon 6 and is therefore predicted to continue translation into exon 7, where it terminates. Public ribosome footprint sequencing data, accessed *via* the GWIPS-*viz* Genome Browser (47), indicate that exon 6 is translated at a substantially lower level than all other exons, and that exon 7 is also substantially translated (Fig. S15*A*), as would be expected if eEF1A-KMT2-207 is the predominantly translated isoform. Public proteomic data from PeptideAtlas (48) also indicate greater protein-level evidence for eEF1A-KMT2-207 than for eEF1A-KMT2-201 (Fig. S15*B*). Only one peptide uniquely mapping to eEF1A-KMT2-201 has been reported in PeptideAtlas, and this is a non-tryptic peptide from immunopeptidomic analyses. On the other hand, several peptides unique to eEF1A-KMT2-207 have been reported in public datasets. In particular, peptides with sequence EELLNEFSEGFELLEELPTPK and SGNSVAALVFQK are frequently detected; these peptides map to the exon 5-exon 7 boundary and to exon 7, respectively (Fig. 5*A*). By comparison with MS/MS spectra we generated from tryptic digestion of recombinantly produced and purified eEF1A-KMT2-207 (see below), we could confirm the identity of these peptides in several existing datasets, including of RKO cells, embryonic stem cells, K562 cells and HEK293 cells (Figs. 5*B* and S16). Moreover, the SGNSVAALVFQK peptide has been detected in many different tissues and cell types, including the pancreas, ovary, testis, tonsil, liver, heart, skin and lymphocytes (49, 50, 51, 52). By considering the lines of evidence above, we conclude that eEF1A-KMT2-207 is the predominantly produced isoform of eEF1A-KMT2 in human cells.

**Figure 6:**
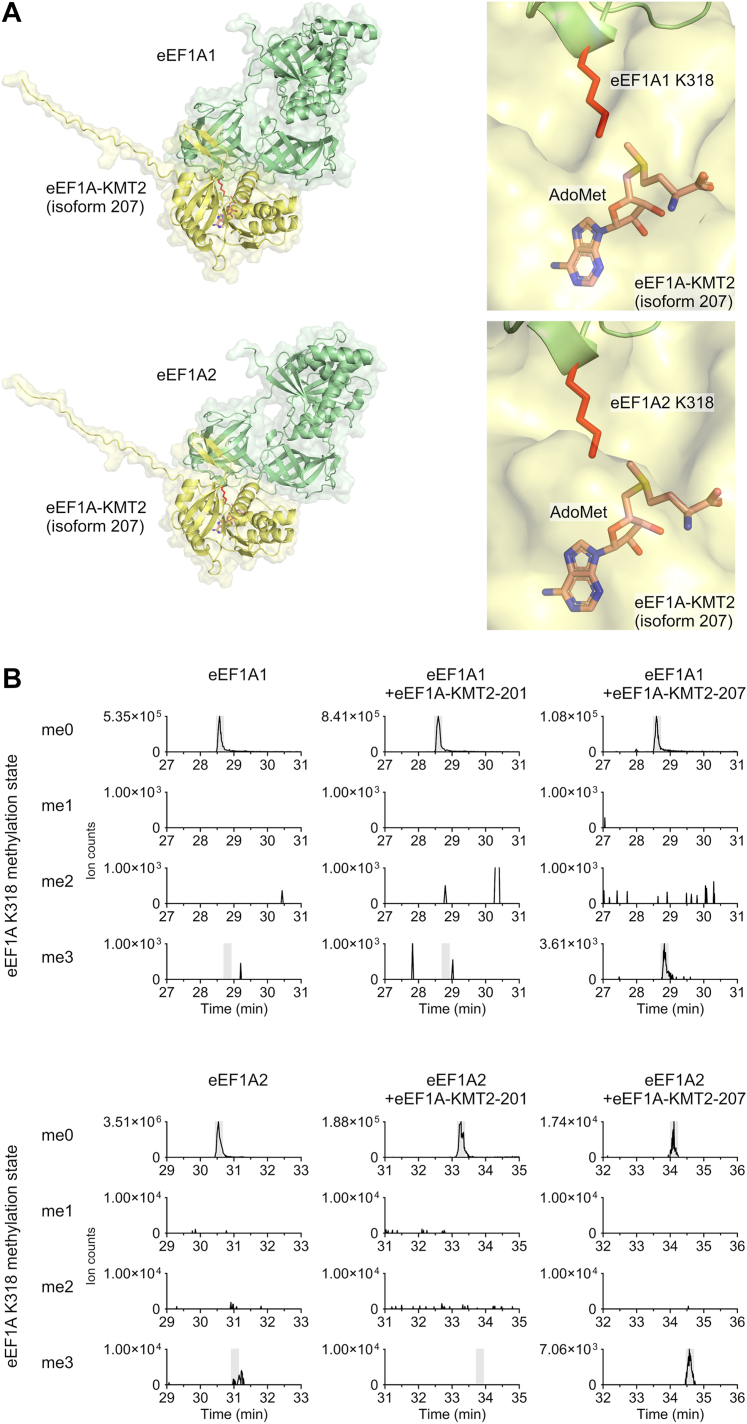
**eEF1A-KMT2 isoform 207 methylates eEF1A1 and eEF1A2 *in vitro*.***A*, *left*: top-ranked AlphaFold-Multimer models of eEF1A-KMT2-207:eEF1A1 and eEF1A-KMT2-207:eEF1A2 complexes shown as cartoon and surface. *Right*: eEF1A1/2 K318 is bound proximal to the predicted AdoMet-binding site of eEF1A-KMT2-207. AdoMet is shown as *sticks*, K318 is shown as *red sticks*, and eEF1A-KMT2-207 is shown as *yellow*, semi-transparent surface. *B*, eEF1A-KMT2-207 methylates eEF1A1 and eEF1A2 at K318 *in vitro*, while eEF1A-KMT2-201 does not. Purified eEF1A1 or eEF1A2 (2.2 μM) were incubated without any enzyme or with eEF1A-KMT2-201 or eEF1A-KMT2-207 (3 μM) in the presence of AdoMet for 18 h at 37 °C. Proteins were separated by SDS-PAGE (see Fig. S20), and eEF1A1 and eEF1A2 gel bands were then digested by AspN and analyzed by LC-MS/MS. Shown are extracted ion chromatograms (XICs) for the triply-charged peptide DNVGFNVKNVSVK (K318 underlined) in its un-, mono-, di-, or tri-methylated states.

AlphaFold-Multimer models of human eEF1A-KMT2-207 bound to either eEF1A1 or eEF1A2 were very similar to each other (Fig. 6*A*). This is not surprising given that eEF1A1 and eEF1A2 are 90% identical in sequence and almost none of the differing residues are found at the predicted interface with eEF1A-KMT2-207 (Fig. S17). Importantly, both predictions showed eEF1A1/2 K318 bound at the active site, proximal to the donated methyl group from AdoMet (Fig. 6*A*). This was as seen for the Efm4:eEF1A complex. *In vitro* methyltransferase activity of human eEF1A-KMT2 has not been demonstrated to date, and mouse eEF1A-KMT2, which has been shown to active *in vitro*, has a more similar C-terminal region to eEF1A-KMT2-207 than to eEF1A-KMT2-201 (Fig. S18) We therefore tested whether either human eEF1A-KMT2-201 or eEF1A-KMT2-207 could methylate human eEF1A1 and eEF1A2 *in vitro*. We had previously seen that human eEF1A1 and eEF1A2 expressed in WT yeast were methylated at K318 (53) (Fig. S19), presumably by Efm4. eEF1A1 and eEF1A2 for use here were thus overexpressed and purified from a ΔEFM4 yeast strain. eEF1A-KMT2-201 and eEF1A-KMT2-207 were expressed and purified from *E. coli*. Purified eEF1A1 or eEF1A2 was then incubated with eEF1A-KMT2-201 or eEF1A-KMT2-207, or without either enzyme, in the presence of AdoMet (Fig. S20), and the methylation status of K318 measured by LC-MS/MS. In the absence of either enzyme eEF1A1 and eEF1A2 were both completely unmethylated at K318, which was expected given the absence of the EFM4 gene in the expression strain. The addition of eEF1A-KMT2-207 led to trimethylation of both eEF1A1 and eEF1A2 at K318 (Fig. 6*B*). In contrast, we observed no methylation in the presence of eEF1A-KMT2-201 (Fig. 6*B*). eEF1A-KMT2-207 therefore catalyzes eEF1A1/2 K318 methylation *in vitro*, while eEF1A-KMT2-201 could not. Combined with the above expression data (Figs. 5 and S15), we conclude that eEF1A-KMT2-207 is the canonical isoform in the human cell and is responsible for methylating eEF1A at K318.

### Mechanisms of substrate recognition are conserved in human eEF1A-KMT2

**Figure 7:**
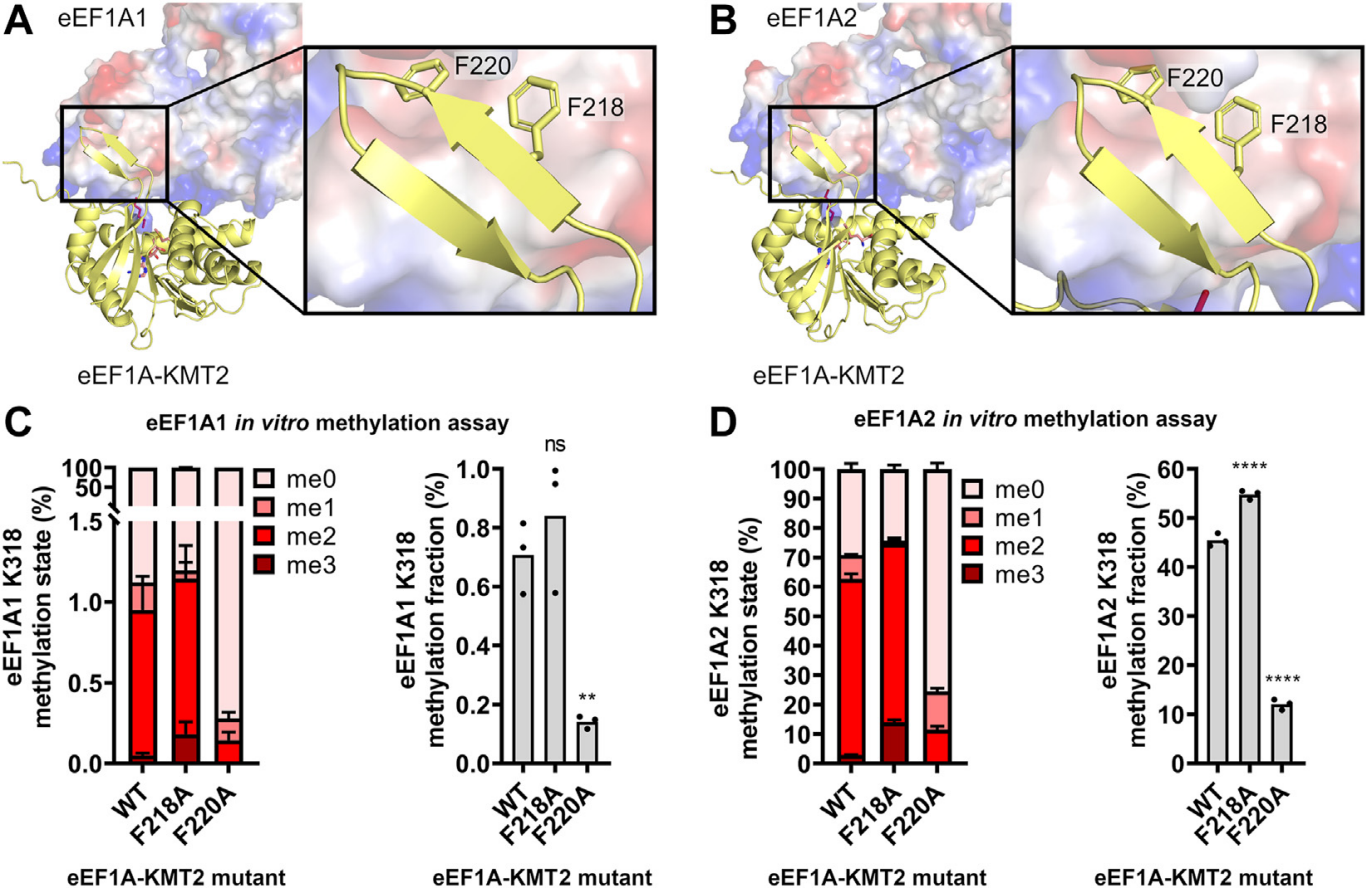
**A conserved phenylalanine in human eEF1A-KMT2 is critical for its eEF1A K318 methylation activity.***A* and *B*, AlphaFold-Multimer model showing a beta-hairpin extending from eEF1A-KMT2 (isoform 207) binding a hydrophobic pocket in domain 2 of eEF1A1 (*A*) or eEF1A2 (*B*). eEF1A-KMT2-207 (*yellow*) is shown as a cartoon structure. eEF1A1/2 is shown as its surface electrostatic potential (*blue* = positive, *red* = negative, *white* = neutral). Inset: sidechains of conserved eEF1A-KMT2-207 residues F218 and F220 on its beta-hairpin are shown as *sticks*. *C* and *D*, *in vitro* methylation assays of eEF1A-KMT2 mutants. Purified WT and mutant eEF1A-KMT-207 (3 μM) were incubated with eEF1A1 (*C*) or eEF1A2 (*D*) (2.2 μM) in the presence of AdoMet for 2 h at 37 °C. Proteins were separated by SDS-PAGE (see Fig. S21), eEF1A1/2 gel bands digested with AspN, and the resulting eEF1A1/2 K318 methylation was detected by LC-MS/MS and quantification of AspN-generated peptide DNVGFNVKNVSVK (K318 underlined) in its triply-charged state. *Left*: Relative levels of eEF1A1/2 K318 methylation states. *Right*: eEF1A1/2 K318 methylation fraction relative to 100% trimethylated K318. Methylation fractions from mutant eEF1A-KMT2 were compared to WT eEF1A-KMT2 using an ordinary one-way ANOVA with a post hoc Dunnett’s multiple comparisons test (ns: not significant, ∗∗*p* ≤ 0.01, ∗∗∗∗*p* ≤ 0.0001).

Our AlphaFold-Multimer models of eEF1A-KMT2-207 bound to eEF1A1 or eEF1A2 revealed that a short beta-hairpin, at positions 217 to 226 and homologous to the beta-hairpin in yeast Efm4, was predicted to bind the same hydrophobic pocket in eEF1A domain 2 (Fig. 7, *A* and *B*). Residues F218 and F220 on this beta-hairpin (homologous to F210 and F212 in yeast Efm4, respectively) were predicted to bind in similar ways to that seen for Efm4 (Fig. 7, *A* and *B*, insets). We therefore tested the effect of alanine mutations of both F218 and F220 on the *in vitro* activity of eEF1A-KMT2-207. Wild-type, F218A or F220A eEF1A-KMT2-207 were incubated with eEF1A1 or eEF1A2 in the presence of AdoMet (Fig. S21), and the resulting K318 methylation was measured by LC-MS/MS. We found that the F220A mutation significantly reduced eEF1A-KMT2-207 methylation of eEF1A K318 (Fig. 7, *C* and *D*), in agreement with it being homologous to F212 in Efm4. The F218A mutation, however, had no or a slightly positive effect on eEF1A-KMT2-207 methylation of eEF1A K318 (Fig. 7, *C* and *D*), indicating that it is not as critical for the activity of the human methyltransferase as it is for the yeast enzyme. Overall, our data show that a conserved phenylalanine in Efm4/eEF1A-KMT2 (F212 and F220, respectively) is critical for its methylation of eEF1A K316/K318 in yeast and human respectively.

### Phosphorylation proximal to eEF1A K316 disrupts Efm4 methyltransferase activity

**Figure 8:**
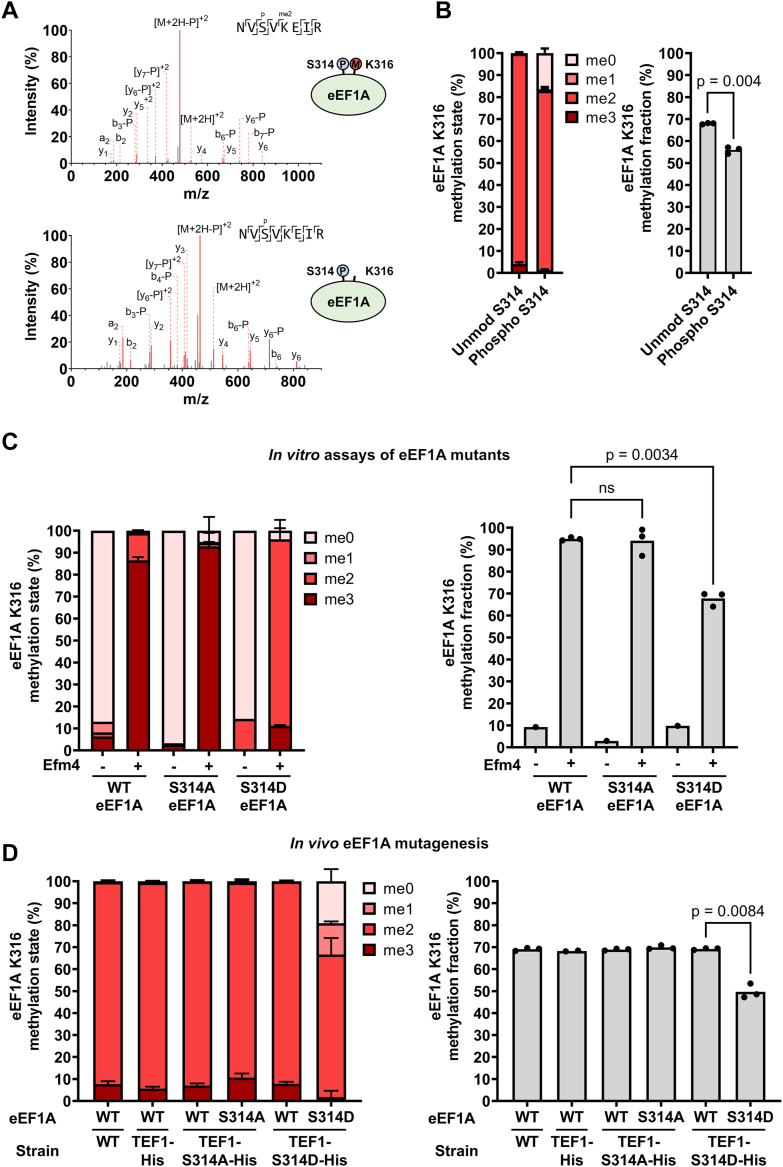
**eEF1A S314 phosphorylation inhibits Efm4-mediated K316 methylation *in vitro* and *in vivo*.***A*, Orbitrap MS/MS spectra of the doubly-charged peptides NVS_p_VK_me2_EIR and NVS_p_VKEIR, indicating eEF1A S314 phosphorylation with and without comodification by K316 dimethylation. Matched fragment ions are *red* and unmatched fragment ions are *gray*. ‘-P’ indicates phosphate neutral loss (-H_3_PO_4_). *B*, phosphorylated S314 correlates with lower methylation levels of K316. Biological triplicate eEF1A purifications were digested by trypsin and subject to phospho-peptide enrichment and analysis by LC-MS/MS. K316 methylation levels on the unphosphorylated peptide NVSVKEIR was measured in the unenriched sample, while K316 methylation levels on the phosphorylated peptide NVSpVKEIR was measured in the enriched sample. Peptide quantities were determined by taking the area under the curve of extracted ion chromatograms (XICs) of the doubly-charged form of the peptide NVSVKEIR in all its methylated/phosphorylated states. *C*, purified WT and mutant eEF1A (2 μM) were incubated with or without purified Efm4 (3 μM) in the presence of AdoMet at 30 °C for 30 min. Assays were carried out in triplicate. Proteins were separated by SDS-PAGE (see Fig. S22), and eEF1A gel bands were digested with trypsin. *D*, chromosomally incorporated eEF1A S314 phospho-mimic mutations reduce K316 methylation levels *in vivo*. Yeast strains were cultured in triplicate for the analysis of K316 methylation, with the except of TEF1-His which was cultured in duplicate. For both (*C*) and (*D*), eEF1A K316 methylation was determined by LC-MS/MS and quantification of tryptic peptides NVSVKEIR (WT eEF1A, K316 underlined), NVAVKEIR (S314A eEF1A, K316 underlined), or NVDVKEIR (S314D eEF1A, K316 underlined) in their doubly-charged state. *Left*: Relative levels of eEF1A K316 methylation states. *Right*: eEF1A K316 methylation fraction relative to 100% trimethylated K316. *p*-values are from two-tailed t-tests without equal variance. ns: not significant.

Our results have shown that N184, a residue on the core methyltransferase fold, is important for Efm4 activity (Fig. 3*C*). N184 is predicted to form hydrogen bonds with eEF1A S314 (Fig. 3*B*), and interestingly, S314 has been reported to be phosphorylated (54). This raises the possibility that S314 phosphorylation might inhibit K316 methylation by Efm4, and thus be an example of negative PTM crosstalk. We therefore sought to confirm and analyze this phosphorylation site and its effect on K316 methylation. To analyze eEF1A phosphorylation *in vivo*, we purified chromosomally hexahistidine-tagged eEF1A from a yeast strain wherein the other copy of eEF1A was deleted (strain TEF1-His ΔTEF2, see Table 1). Purified eEF1A was then digested by trypsin and phosphorylated peptides were enriched using titanium dioxide. Total peptides, not subject to phosphopeptide enrichment, were also analyzed. In the phosphopeptide-enriched samples, we detected a tryptic peptide containing both S314 phosphorylation and K316 dimethylation (NVS_p_VK_me2_EIR, Fig. 8*A*), confirming that these modifications can co-occur on eEF1A *in vivo*. Interestingly, we also detected a form of this peptide with S314 phosphorylation but without K316 methylation (NVS_p_VKEIR, Fig. 8*A*). Quantification of K316 methylation states when S314 is phosphorylated (in the phosphopeptide-enriched samples) and when S314 is unmodified (in the unenriched samples) revealed a significant decrease in K316 methylation when S314 is phosphorylated (Fig. 8*B*) This suggests that S314 phosphorylation negatively impacts the deposition of K316 methylation.

To further investigate whether S314 phosphorylation directly inhibits deposition of K316 methylation, we tested the effect of S314 phospho-null (S314A) and phospho-mimic (S314D) mutations on K316 methylation by Efm4 *in vitro* and *in vivo*. We first tested whether these phospho-mutants affect Efm4 activity toward eEF1A *in vitro*. WT, S314A and S314D eEF1A were purified from a ΔEFM4 strain and incubated with or without purified Efm4 in the presence of AdoMet (Fig. S22). The levels of K316 methylation were then analyzed by LC-MS/MS. Controls without Efm4 showed minimal methylation levels (Fig. 8*C*). While Efm4 methylated both WT and S314A eEF1A to the same degree, S314D eEF1A was significantly less methylated compared with WT and S314A eEF1A (Fig. 8*C*). Specifically, the S314D mutation resulted in a ∼28% decrease in K316 methylation relative to WT, mostly due to a substantial shift from predominantly trimethylated K316 for WT eEF1A to predominantly dimethylated K316 for S314D eEF1A (Fig. 8*C*). We then tested the effect of genomic S314 phospho-mutants on eEF1A K316 methylation *in vivo*. For this mutagenesis, the fact that eEF1A is encoded by two genes, TEF1 and TEF2, was used to enable direct comparison of WT and mutant eEF1A within the same yeast strain. TEF1 was mutated to encode either S314A or S314D eEF1A, in two different strains, while TEF2 was retained as encoding WT eEF1A in both strains. Importantly, it was possible to quantify the methylation of TEF1-encoded S314A or S314D eEF1A separately from the TEF2-encoded WT eEF1A since they generate distinct and distinguishable K316-containing peptides. A hexahistidine tag was also added to the end of TEF1 gene, which we confirmed did not affect K316 methylation levels (Fig. 8*D*, WT *versus* TEF1-His). We also confirmed that S314A and S314D mutations did not affect eEF1A expression from the TEF1 gene, as an immunoblot against the hexahistidine tag showed comparable levels of hexahistidine-tagged eEF1A across strains (Fig. S23). Mass spectrometric analysis revealed that the S314D mutation significantly reduced K316 methylation by ∼28% (Fig. 8*D*, TEF1-S314D-His: WT *versus* S314D), while the S314A mutation did not affect K316 methylation levels (Fig. 8*D*, TEF1-S314A-His: WT *versus* S314A). These results demonstrate that a phospho-mimic mutation of eEF1A S314 results in a significant decrease in Efm4-catalyzed methylation at K316. Conversely, a phospho-null mutation of S314 has no measurable effect on K316 methylation, likely due to the low stoichiometry of the S314 phosphorylation event. Overall, we have shown that phosphorylation of S314 crosstalks with K316 methylation, and that this occurs through S314 phosphorylation-mediated inhibition of Efm4 catalytic activity towards K316. Phosphorylation of S314 likely disrupts hydrogen bonding to Efm4 N184 and/or causes steric hindrance with Efm4 L149 and N184 (Fig. S24). Notably, this is the first evidence of PTM crosstalk on eEF1A.

## Discussion

Many yeast and human protein methyltransferases targeting just a single amino acid in a single protein in the cell have been discovered in the last 2 decades (10, 55, 56). Structural studies have shown that the specificity of these enzymes is often facilitated by extensive networks of contacts between the methyltransferase and its substrate protein, or in the case of histone methyltransferases, the nucleosome (15, 57, 58, 59). Here, we have shown how a small methyltransferase can achieve substrate specificity by taking advantage of unique features of the substrate proximal to the target methylation site. Specifically, we elucidated how a highly conserved eukaryotic lysine methyltransferase recognizes and methylates translation elongation factor 1A (eEF1A). We showed that a conserved beta-hairpin extending from the core methyltransferase fold is important for yeast Efm4 methylation of eEF1A K316 *in vitro*, and that two conserved phenylalanines on this beta-hairpin, F210 and F212, are important for Efm4 methylation of eEF1A both *in vitro* and *in vivo*. We further showed that mutation of F220 in the human enzyme eEF1A-KMT2 (equivalent to F212 in Efm4) significantly reduced its methylation of eEF1A1 and eEF1A2 *in vitro*. Notably, the eEF1A hydrophobic pocket bound by F212/F220 in Efm4/eEF1A-KMT2 is also the binding site for eEF1Bα F163 and the 3′ terminal adenosine of aa-tRNA (25, 27). In agreement with this, we showed that eEF1Bα inhibits Efm4 methylation of eEF1A K316 *in vitro*, and that a F163A mutation of eEF1Bα reduces this inhibition.

eEF1A is targeted by the second largest number of dedicated methyltransferases in the eukaryotic cell after histone H3 (29). Each methyltransferase methylates a different eEF1A amino acid, yet the mechanisms underpinning this specificity have been poorly understood. Two eEF1A methyltransferases, the human 7βS methyltransferase METTL13 (C-terminal domain) and the yeast SET domain methyltransferase Efm1, recognize specific linear sequence motifs and thereby specifically methylate eEF1A at its N-terminus and K30, respectively (13, 60). Here we have shown that eEF1A methyltransferases, Efm4 and eEF1A-KMT2, recognize three-dimensional topological features of eEF1A distal in sequence to their target sites. Given that almost all other yeast and human eEF1A methyltransferases are small 7βS enzymes, they may also recognize their eEF1A methylation sites through similar mechanisms. Deciphering how these unique methyltransferases recognize and methylate eEF1A will be important for understanding how they can be targeted therapeutically. This is important as differential expression of eEF1A methyltransferases is associated with cancer progression (61, 62, 63, 64). For example, increased eEF1A K55 methylation by overexpression of METTL13 plays a central role in the development of lung and pancreatic cancers (63). Conversely, reduced expression of eEF1A-KMT2 is correlated with decreased survival in patients with renal cell carcinoma (62), while lower expression of eEF1A-KMT3 in gastric cancer is associated with poor prognosis (61). Separate to cancer, a decrease in eEF1A methylation levels in muscle tissue may be associated with aging (65). In addition to their highly specific nature, eEF1A methyltransferases also come from diverse evolutionary lineages (29), and may therefore be amenable to methyltransferase-specific drug targeting. For all these reasons, eEF1A methyltransferases are attractive candidates for therapeutic investigation, and understanding the mechanisms underpinning their substrate recognition is critical for this.

Our results provide insights into how eEF1A methyltransferases specifically methylate their essential substrate protein (29). However, the function of eEF1A methylation remains unclear. Extensive analyses have revealed little to no effect of the absence of methylation on eEF1A molecular function or interactions (60, 66, 67, 68). In particular, loss of any individual methylation site does not affect eEF1A interaction with eEF1Bα or other members of the eEF1 complex (60, 68). One exception is that the loss of K55 methylation in human eEF1A modestly decreases its intrinsic GTPase activity (63). Despite this, several cell growth phenotypes and codon-selection biases associated with eEF1A methyltransferase knockouts have been described (60, 66, 69). In particular, eEF1A K316 methylation was reported to be important for viral replication (70). Given that Efm4 and eEF1A-KMT2 appear to bind the same pocket in eEF1A domain 2 bound by eEF1Bα and aa-tRNA, an interesting possibility is that Efm4/eEF1A-KMT2 binding and subsequent methylation of K316/K318 serves as a mark of eEF1A that is fully folded and competent for translation. Investigations combining loss of this methylation site with perturbation of eEF1A biogenesis, for example by disruption of the eEF1A-specific chaperone Zpr1 (71), may provide insight into the role of this modification in eEF1A functions and protein synthesis.

Crosstalk between post-translational modifications (PTMs) is a key way the cell integrates information to enact specific cellular outcomes (72). As eEF1A is known to be highly post-translationally modified, it has been proposed that an ‘eEF1A code’ might regulate eEF1A function through the generation of distinct pools of eEF1A which carry different subsets of PTMs (29, 30, 73, 74). These different populations of eEF1A molecules may be involved in translation elongation or may be involved in other non-canonical functions (21). One core aspect of PTM codes is crosstalk between modifications, whereby the presence or absence of a particular PTM affects the presence or absence of another PTM (75). Methylation and phosphorylation, in particular, are often seen to engage in crosstalk with each other (76, 77). Here we report the first instance of PTM crosstalk on eEF1A. In particular, we showed that phosphorylation at S314 partially inhibits nearby K316 methylation by Efm4. It remains unclear whether K316 methylation can affect deposition of S314 phosphorylation and this question has not been investigated here as the cognate kinase is unknown. However, given that the reduction in *in vivo* K316 methylation in the S314 phospho-mimic mutant was similar to that seen in the presence of S314 phosphorylation (Fig. 8, *B* and *D*), it seems likely that this is a monodirectional crosstalk, whereby S314 phosphorylation is unaffected by K316 methylation. Although the reduction in K316 methylation upon S314 phosphorylation is modest in relative terms, since eEF1A is one of the most abundant proteins in the cell, this represents a significant population of eEF1A molecules that are being modulated. Importantly, since there are no known eEF1A demethylases, this crosstalk necessarily requires S314 phosphorylation prior to K316 methylation. As this crosstalk appears to be monodirectional, its function will be linked to the role of K316 methylation, which remains unknown. Future work should investigate the individual and combined effects of loss of S314 phosphorylation and loss of K316 methylation to unravel the role of S314 phosphorylation-mediated inhibition of K316 methylation. Since eEF1A is highly methylated and phosphorylated, further investigations should also probe whether there are other instances of phospho-methyl crosstalk on this essential protein.

Deep learning methods like AlphaFold and RoseTTAFold have proved successful for predicting the structures of protein complexes (78, 79, 80). Our results demonstrate that they may be particularly useful for the prediction of enzyme-substrate interactions for protein modifications, especially as these transient interactions are difficult to capture in co-crystal structures. The highly specific nature of many methyltransferase-substrate interactions makes them particularly amenable to predictive approaches, as these interactions necessarily involve a specific target residue positioned in the active site of the methyltransferase. This correct positioning provides a clear way to triage models. In fact, such triaging led us to discover that a secondary isoform of eEF1A-KMT2 is responsible for eEF1A K318 methylation. Notably, a recent study employed AlphaFold modeling to successfully predict interfacing residues between the human elongation factor 2 (eEF2) lysine methyltransferase eEF2-KMT/FAM86A and its substrate, eEF2 (18). We therefore anticipate that predictive modeling approaches will be critical for deciphering the mechanisms of enzyme-substrate interactions for protein modifications.

## Experimental procedures

### Structural modeling and visualization

Five models of each complex were generated using AlphaFold-Multimer v3 (78) as implemented in ColabFold v1.5.2 (81), with the MSA generated on the MMSeqs2 server and using AMBER relaxation. Specifically, input parameters were “--model-type AlphaFold2-multimer --recompile-all-models --amber”; see the ColabFold repository (https://github.com/sokrypton/ColabFold) for more information. *S*-adenosyl-L-methionine (AdoMet) was docked into AlphaFold-Multimer models using COACH-D (82). Models and structures were visualized and aligned in PyMOL v2.5.2 with the “align” command. In all cases, structures and models were aligned relative to eEF1A domains 2 and 3, as these represent a single structural unit. Models can be found in the Supporting Information.

### Generation of yeast strains and culturing

All *S. cerevisiae* strains were generated in the background of BY4741 (see Table 1). C-terminally hexahistidine-tagged TEF1 or EFM4 were amplified from plasmids BG1805-TEF1 and BG1805-EFM4, respectively. These were then cloned into the pRS426 plasmid upstream of URA3 by Gibson assembly. Site-directed mutagenesis of pRS426-TEF1 or pRS426-EFM4 was then carried out using mutagenic primers, as described previously (83). Wild-type and mutant TEF1 and EFM4 genes, along with the URA3 selection marker, were amplified from pRS426 plasmids incorporated into the genomes of WT or ΔEFM4 yeast through homologous recombination, according to previous methods (84). TEF2 was deleted from the TEF1-His strain through replacement with the clonNAT resistance cassette *natNT2* according to previous methods (85).

Yeast were cultured in YEPD (1% w/v yeast extract, 2% w/v peptone, 2% w/v glucose) to mid-log phase (OD_600_ ∼0.8), harvested by centrifugation (4500*g*, 4 °C, 5 min) and stored at −80 °C until lysis. For generation of human eEF1A1 and eEF1A2, pD1204-eEF1A1 and pD1204-eEF1A2, as cloned previously (53), were transformed into the ΔEFM4 yeast strain and overexpressed as described previously (84).

### Yeast lysis and protein purification

For purification of yeast eEF1A and human eEF1A1 and eEF1A2, yeast cells were resuspended in yeast His-tag lysis buffer (50 mM sodium phosphate, 500 mM NaCl, 40 mM imidazole, 20% (v/v) glycerol, 0.25% (v/v) Triton X-100, pH 8.0) with 1× cOmplete EDTA-free Protease Inhibitor Cocktail. For analysis of eEF1A phosphorylation, 1× PhosSTOP phosphatase inhibitor was also included. Cell were lysed by 3 to five rounds of bead-beating for 1 min each before lysates were clarified by centrifugation (21,000*g*, 4 °C, 40 min). Hexahistidine-tagged eEF1A was then purified from lysates using 1 ml Ni-NTA Superflow Cartridges (Qiagen). Cartridges were first equilibrated with 10 ml of yeast His-tag lysis buffer, before clarified lysate was applied and cartridges washed with 10 ml of yeast His-tag lysis buffer followed by 10 ml of His-tag purification buffer (50 mM sodium phosphate, 500 mM NaCl, 40 mM imidazole, 20% glycerol, pH 8). eEF1A was then eluted with 3 to 4 ml of His-tag elution buffer (50 mM sodium phosphate, 500 mM NaCl, 500 mM imidazole, pH 7.4) and subsequently buffer-exchanged into 50 mM sodium phosphate/200 mM NaCl (pH 7.4) with Amicon Ultra-4 Centrifugal Filter Units with Ultracel-3 membrane.

### Cloning, expression and purification of recombinant proteins

EFM4, EEF1AKMT2-201, EEF1AKMT2-207 and EFB1 (eEF1Bα) were cloned into pET15b by Gibson assembly for subsequent bacterial expression. EFM4 containing the upstream region (*i.e.* chrIX:241,943-242,716) was amplified from WT (BY4741) yeast genomic DNA, using primers to insert a C-terminal hexahistidine tag. N-terminally hexahistidine-tagged EFB1 and C-terminally hexahistidine-tagged EEF1AKMT2-201 and EEF1AKMT2-207 were obtained as gBlocks (Integrated DNA Technologies). pET15b-EFM4 without the upstream region of EFM4 was generated by site-directed ligase-independent mutagenesis (SLIM) (86). Point mutations of pET15b-EFM4, pET15b-EEF1AKMT2-207 and pET15b-EFB1 were generated using mutagenic primers, as described previously (83). pET15b plasmids were then transformed into Rosetta (DE3) *E. coli* and cells were grown in lysogeny broth (LB) at 37 °C to an OD_600_ of ∼0.6, before expression was induced by 1 mM IPTG and cultures were grown for 5 h at 25 °C. Cells were harvested by centrifugation (4500*g*, 4 °C, 10 min) and pellets stored at −80 °C until purification.

*E. coli* cells were resuspended in His-tag purification buffer with 1× cOmplete EDTA-free Protease Inhibitor Cocktail. Cells were lysed by 3 rounds of sonication with an ultrasonic probe (40% amplitude for 30s, alternating 0.5 s on/0.5 s off), with 3 min of cooling in ice-water between runs, and subsequent lysates were clarified by centrifugation (21,000*g*, 4 °C, 40 min). Hexahistidine-tagged proteins were then either purified with 1 ml Ni-NTA cartridges, as for eEF1A above, or with His Mag Sepharose Ni resin (Cytiva). For His Mag purifications, 100 μl of beads were pre-equilibrated with His-tag purification buffer before incubation with clarified lysate (30 min, room temperature, 1000 rpm). Beads were washed three times with 500 μl His-tag purification buffer before eluting twice by incubating with 100 μl His-tag elution buffer for 10 min at 15 °C, with shaking at 1000 rpm. Elutions were then buffer-exchanged into 50 mM sodium phosphate/200 mM NaCl (pH 7.4) with Amicon Ultra-0.5 Centrifugal Filter Units with Ultracel-3 membrane.

### DSSO crosslinking reaction

Purified Efm4 (16.4 μM, ∼25 μg total) and eEF1A (from strain ΔEFM4 TEF1-His, 3.75 μM, ∼11 μg total) were incubated in the presence of AdoMet (50 μM) and DSSO (1 mM) in *in vitro* methylation buffer (50 mM HEPES, 20 mM NaCl, 1 mM EDTA, pH 7.4) in a total volume of 60 μl at 30 °C for 1 h. Crosslinking was terminated by the addition of Tris-Cl (pH 8.0) to 10 mM. The sample was then split in three and buffer-exchanged into either 50 mM NH_4_HCO_3_, for trypsin and GluC digests, or 100 mM Tris-Cl (pH 8.0)/10 mM CaCl_2_, for the chymotrypsin digest. Proteins were reduced with 10 mM DTT for 1 h at 37 °C and alkylated with 15 mM iodoacetamide (IAA) for 1 h at room temperature. Proteins were then digested with either 400 ng sequencing grade trypsin (Promega), 800 ng Sequencing Grade GluC (Promega), or 800 ng Chymotrypsin (Promega), at either 37 °C (trypsin and GluC) or 25 °C (chymotrypsin) for 18 h. Digested peptides were then desalted with 50 mg tC18 Sep-Pak columns (Waters) and resuspended in 0.1% formic acid. Crosslinked peptides were analyzed on an Orbitrap Fusion Lumos Tribrid mass spectrometer (Thermo Scientific) as described below.

### *In vitro* methylation assays

Purified yeast or human eEF1A were incubated with or without purified methyltransferases (Efm4, eEF1A-KMT2-207 or eEF1A-KMT2-201) in *in vitro* methylation buffer in the presence of 500 μM AdoMet at 30 °C (yeast) or 37 °C (human) for the indicated times. Reactions were terminated by the addition of 6× SDS loading buffer (350 mM Tris-Cl, 30% (v/v) glycerol, 10% (w/v) SDS, 600 mM DTT, 0.012% (w/v) bromophenol blue) and boiling for 10 min. Proteins were separated by SDS-PAGE on NuPAGE 4 to 12% Bis-Tris gels with MES SDS running buffer, fixed with 25% isopropanol/10% acetic acid and stained with QC Colloidal Coomassie Stain (Bio-Rad). Gels were imaged under white light using a ChemiDoc XRS+ Imaging System (Bio-Rad). Gel bands corresponding to eEF1A were excised, digested with either 35 to 50 ng AspN or 50 ng trypsin and prepared for mass spectrometry according to previous methods (36). Samples were analyzed on either an Orbitrap Fusion Lumos Tribrid mass spectrometer (Thermo Scientific) or an LTQ Orbitrap Velos mass spectrometer (Thermo Scientific), as detailed below.

### Analysis of eEF1A methylation in EFM4 mutant strains

All clones of yeast strains EFM4-His, EFM4-F210A-His, EFM4-F212A-His were grown to mid-log phase (OD_600_ ∼0.8) in YEPD, cells harvested by centrifugation (4500*g*, 4 °C, 5 min) and resuspended in HEPES lysis buffer (50 mM HEPES, 100 mM NaCl, 0.5% Triton X-100, 2 mM DTT, 2 mM EDTA, pH 7.5, 1× cOmplete EDTA-free Protease Inhibitor Cocktail). Cells were lysed by bead-beating and clarified as described above. Approximately 50 μg of protein from each lysate was then buffer-exchanged into 50 mM NH_4_HCO_3_, reduced with 10 mM DTT for 30 min at 37 °C and alkylated with 15 mM IAA for 30 min at room temperature, before digestion with 1 μg of Sequencing Grade GluC (Promega) for 18 h at 37 °C. Digested peptides were then desalted with 50 mg tC18 Sep-Pak columns (Waters) and resuspended in 0.1% formic acid.

To quantify eEF1A K316 methylation levels in EFM4 mutant strains, a GluC peptide containing K316 (QGVPGDNVGFNVKNVSVKE, K316 unlined) was analyzed by parallel reaction monitoring (PRM) on an Orbitrap Fusion Lumos Tribrid mass spectrometer (Thermo Scientific). Peptides were separated by nanoLC and ionized by ESI, as described previously (87). Then, PRM analysis consisted of a precursor scan acquired in the Orbitrap (350–1500 m/z, resolution = 60,000), followed by four MS/MS scans targeting the triply-charged GluC peptide in its un-, mono-, di- and tri-methylated state (*m/z* = 663.01, 667.68, 672.36 and 677.03) also acquired in the Orbitrap (resolution = 30,000, HCD NCE = 30, isolation width = 1.5 *m/z*). Data were analyzed in Skyline v21.2.0.568 and eEF1A K316 methylation was quantified using the area under the curve of singly-charged fragment ions b7, b8, b9, b10, b11, b12, b13, b14, y6, y7, y8, y9, y10, y11, y12 and y13.

For analysis of Efm4 levels in yeast strains EFM4-His, EFM4-F210A-His and EFM4-F212A-His, cultures of one clone of each strain, as well as the background strain BY4741, were grown to mid-log and cells harvested, as described above. Cells were resuspended in His-tag lysis buffer, lysed by bead-beating as described above, and hexahistidine-tagged Efm4 was enriched from lysates using His Mag Sepharose Ni resin (Cytiva), as described above. Resulting eluates were separated by SDS-PAGE, the region of the gel corresponding to the size of Efm4 was excised and the resulting gel bands were digested with trypsin, as described above. Samples were then analyzed on a Q Exactive Plus mass spectrometer (Thermo Scientific), as described previously (36).

### Phospho-peptide enrichment and sample preparation for analysis of eEF1A phosphorylation

Triplicate purifications of eEF1A from strain TEF1-His ΔTEF2 were carried out as described above. For each replicate, approximately 50 μg of purified eEF1A was buffer-exchanged into 50 mM NH_4_HCO_3_, reduced with 10 mM DTT for 1 h at 37 °C and alkylated with 15 mM IAA for 1 h at room temperature. Samples were then digested with 100 ng of sequencing grade trypsin (Promega) for 18 h at 37 °C, before desalting with tC18 Sep-Pak columns (50 mg, Waters). Five percent of each sample was retained, while the remaining 95% was subjected to phospho-peptide enrichment using Titansphere TiO_2_ beads (GL Sciences), according to the modified protocol in reference (87). Phosphopeptide-enriched samples were then analyzed by LC-MS/MS on an Orbitrap Fusion Lumos mass spectrometer as detailed below.

### Analysis of eEF1A methylation in eEF1A phospho-mutant strains

Triplicate cultures of yeast strains TEF1-His, TEF1-S314A-His and TEF1-S314D-His were grown to mid-log phase (OD_600_ ∼0.8) in YEPD, cells harvested and resuspended in HEPES lysis buffer. Cells were lysed by bead-beating, clarified and lysates separated by SDS-PAGE, as described above. Gel bands corresponding to eEF1A (∼50 kDa) were excised and digested with trypsin, as described above. Peptide samples were then analyzed on an Orbitrap Fusion Lumos mass spectrometry as detailed below. Levels of wild-type and mutant eEF1A in these strains were confirmed by immunoblotting against the hexahistidine tag with a Penta-His HRP antibody (Qiagen, cat. no. 34460, lot no. 172032877), as described previously (88). Lysates were also immunoblotted against PGK1 as a loading control, using an anti-3-phosphoglycerate antibody (Molecular Probes, cat. no. A-6457, lot no. 71C1-1), as described previously (88).

### Mass spectrometry

Peptides were separated by nanoLC on a C18 nanocolumn over a 32 min gradient and ionized by ESI, as described previously (87). For crosslinked samples the following gradient was used with solvent A (0.1% formic acid) and solvent B (80% acetonitrile/0.1% formic acid): 0 to 4 min (2–10% B), 4 to 45 min (2–10% B), 45 to 135 min (10–25% B), 135 to 163 (25–80% B), 163 to 165 min (80% B), 165 to 172 min (80% B reducing to 2% B), 172 to 180 min (2% B).

For analysis on the Orbitrap Fusion Lumos, precursor scans were acquired in the Orbitrap (350–1750 *m/z*, resolution = 60,000), before selected precursors were isolated (isolation width = 1.6 *m/z*), fragmented with HCD (NCE = 30) and fragment ions analyzed in the Orbitrap (resolution = 30,000). For analysis of crosslinked samples, precursor scans were acquired in the range 400 to 2000 *m/z*, precursors (charge state 3+ to 8+) were fragmented with stepped HCD (NCE = 21/27/33) and fragment ions analyzed in the Orbitrap (resolution = 60,000). The TopSpeed setting was used to limit the duty cycle to 2 s and dynamic exclusion was set at 20 s.

Analyses on the LTQ Orbitrap Velos mass spectrometer (Thermo Scientific) were carried out according to previous methods (36), except that inclusion lists were not used.

### Data analysis

Raw files were converted to Mascot generic format (mgf) using RawConverter v1.2.0.1. MS/MS spectra were then searched against the SwissProt database (SwissProt 2022_05 (568,744 sequences; 205,548,017 residues)) with a contaminants database included (contaminants 20,090,624: 262 sequences, 133,770 residues), in Mascot v2.8, (Matrix Science), with the following settings: taxonomy: *S. cerevisiae* (Baker’s yeast) or *Homo sapiens* (Human); Enzyme: trypsin or Asp-N_ambic; Missed cleavages: 3 (trypsin samples) or 5 (AspN samples); Peptide tolerance: 4 ppm; MS/MS tolerance: 20 ppm (Orbitrap Fusion Lumos and Q Exactive Plus) or 0.4 Da (LTQ Orbitrap Velos); Instrument: Default; Fixed modifications: Carbamidomethyl (C) (for samples subjected to reduction and alkylation); Variable modifications: Acetyl (Protein N-term), Oxidation (M), Methyl (K), Dimethyl (K), Trimethyl (K), with Phospho (ST) included for S314 phosphorylation-related samples, and Ser->Ala (S) or Ser->Asp (S) included for S314 phospho-mutant samples. For analysis of purified, recombinant eEF1A-KMT2-207, data were further searched against the human expressed sequence tag database, that is, Human_EST 135 (52,233,594 sequences; 8,864,668,104 residues).

eEF1A methylation was quantified by taking the area under the curve of extracted ion chromatograms (XICs) of indicated peptide ions with a tolerance of ±10 ppm in Thermo XCalibur 2.2 SP1.48 Qual Browser. The percentage of each methylation state was determined by dividing its intensity by the sum total of intensities for all methylation states (including unmethylated). The methylation fraction percentage was calculated by summing the methylation state percentages, weighted according to the number of methyl groups (*i.e.* me0% × 0 + me1% × 1 + me2% × 2 + me3% × 3), and dividing by the theoretical max value for full di- or tri-methylation, as detailed previously (13).

XL-MS data were analyzed with MaxLynx (89) as implemented in MaxQuant v2.2.0.0. Data were searched individually for each protease (trypsin, chymotrypsin or GluC) against custom sequence databases consisting of proteins identified in each sample. These were generated by searching data against the SwissProt database in a standard (not crosslinking) search using Mascot (as above). For MaxLynx analyses, “Cross linking type” was set as MS2-cleavable with DSSO selected as the crosslinker and with default settings. Trypsin, GluC and chymotrypsin digests were analyzed separately with the following settings: Trypsin: Protease = Trypsin/P, Max missed cleavages = 3; GluC: Proteases = GluC and AspC, Max missed cleavages = 5; Chymotrypsin: Proteases = Trypsin/P and Chymotrypsin+, Max missed cleavages = 5.

## Data availability

The mass spectrometry proteomics data have been deposited to the ProteomeXchange Consortium *via* the PRIDE (90) partner repository with the dataset identifier PXD042599.

## Supporting information

This article contains supporting information.

## Conflict of interest

The authors declare that they have no conflicts of interest with the contents of this article.
