# Supplementary material for: Methylation of elongation factor 1A by yeast Efm4 or human eEF1A-KMT2 involves a beta-hairpin recognition motif and crosstalks with phosphorylation
Source: J Biol Chem. 2024 Jan 8;300(2):105639. doi: 10.1016/j.jbc.2024.105639 (PMC10844748; doi:10.1016/j.jbc.2024.105639)
Supplement: Supporting Figures [file mmc1.pdf]

Methylation of elongation factor 1A by yeast Efm4 or human eEF1A-KMT2 involves a beta-hairpin recognition motif and crosstalks with phosphorylation

Joshua J. Hamey[1*], Amy Nguyen[1], Mahdi Haddad[1], Xabier Vázquez-Campos[1], Paige G. Pfeiffer[1], Marc R. Wilkins[1]

[1]School of Biotechnology and Biomolecular Sciences, University of New South Wales, NSW, Australia

*Corresponding author: Dr. Joshua J. Hamey, j.hamey@unsw.edu.au

Supporting information

Figure S1. AlphaFold-Multimer models of the Efm4:eEF1A complex.

Figure S2. Efm4 and eEF1A from rank 1 AlphaFold-Multimer model show expected folded domains.

Figure S3. The EFM4 gene begins at the currently annotated M29 position, chrIX:242,027.

Figure S4. Deletion of incorrectly annotated upstream region of Efm4 does not affect its activity *in vitro*.

Figure S5. eEF1A K316 methylation generated during the DSSO crosslinking reaction.

Figure S6. MS/MS spectra identifying DSSO crosslinks between Efm4 and eEF1A.

Figure S7. Coomassie-stained SDS-PAGE gel of Efm4 time series assay.

Figure S8. Coomassie-stained SDS-PAGE gel of Efm4 mutant assays.

Figure S9. Genomic mutations of EFM4 do not substantially affect its expression.

Figure S10. Coomassie-stained SDS-PAGE gel of Efm4 assays with WT and F163A eEF1Bα.

Figure S11. AlphaFold-Multimer models of eEF1A-KMT-201 bound to eEF1A1.

Figure S12. AlphaFold-Multimer models of eEF1A-KMT-201 bound to eEF1A2.

Figure S13. AlphaFold-Multimer models of eEF1A-KMT-207 bound to eEF1A1.

Figure S14. AlphaFold-Multimer models of eEF1A-KMT-207 bound to eEF1A2.

Figure S15. Public mRNA-seq, Ribo-seq and proteomic data indicate that eEF1A-KMT2-207 is the predominantly expressed isoform.

Figure S16. Comparison of MS/MS spectra confirm identity of eEF1A-KMT2-207-specific peptides in cell lines.

Figure S17. Almost all residues unique to eEF1A2 are not at the interface with eEF1A-KMT2.

Figure S18. Mouse eEF1A-KMT2 is more similar to human eEF1A-KMT2-207 than eEF1A-KMT2-201.

Figure S19. Human eEF1A1 and eEF1A2 purified from WT yeast are methylated at K318.

Figure S20. Coomassie-stained SDS-PAGE gel of eEF1A-KMT2-201 and eEF1A-KMT2-207 methylation assays of eEF1A1 and eEF1A2.

Figure S21. Coomassie-stained SDS-PAGE gels of eEF1A-KMT2-207 F218A and F220A mutant assays.

Figure S22. Coomassie-stained SDS-PAGE gels of eEF1A phospho-mutant assays.

Figure S23. PentaHis immunoblot confirms phospho-mutants have no effect on eEF1A protein levels.

Figure S24. Phosphorylated eEF1A S314 likely causes steric clashes with Efm4 L149 and N184.

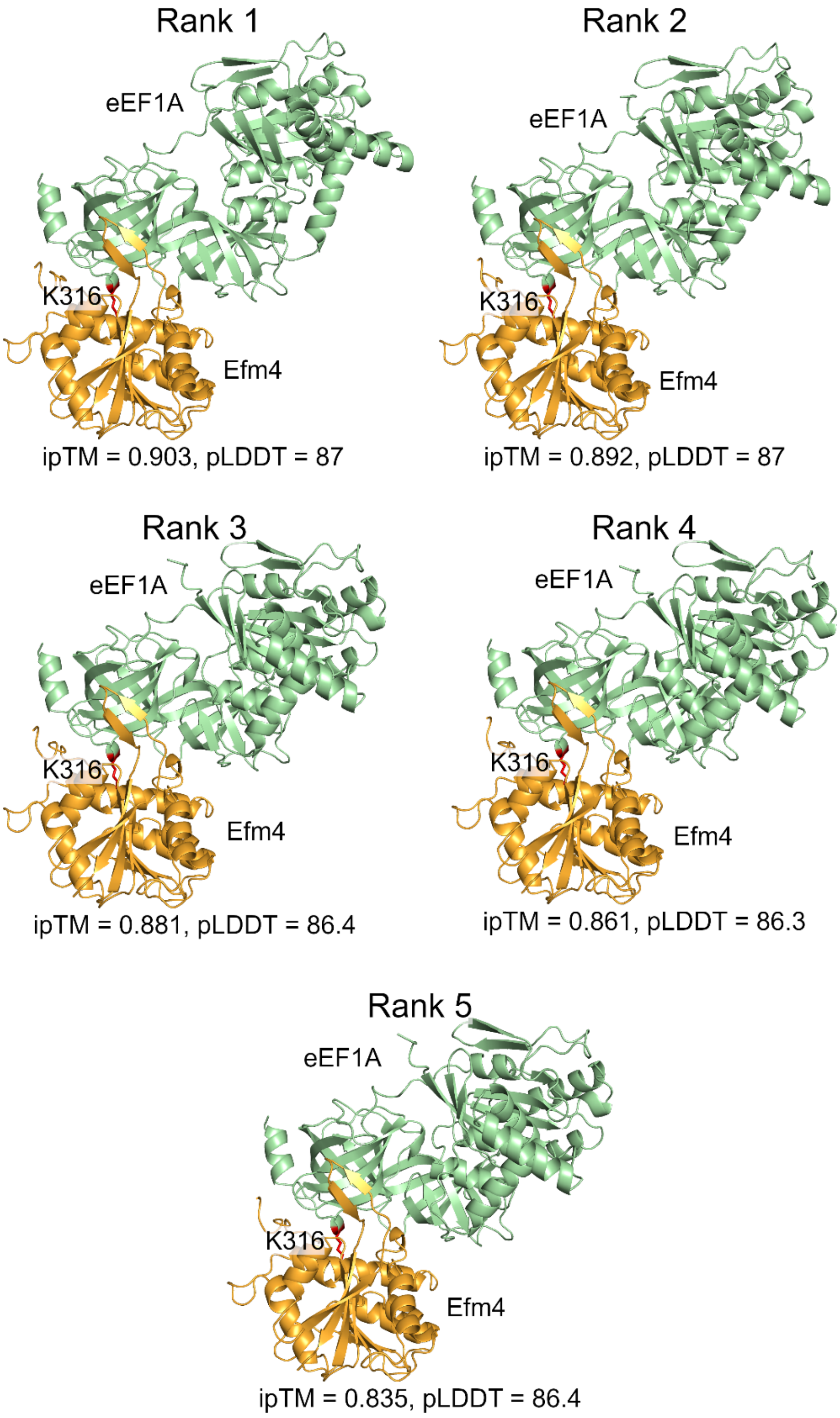

Figure S1. AlphaFold-Multimer models of the Efm4:eEF1A complex.

AlphaFold-Multimer models of Efm4 bound to eEF1A were generated as described in methods. The main variation between models is the conformational flexibility of domain 1 relative to domains 2 and 3.

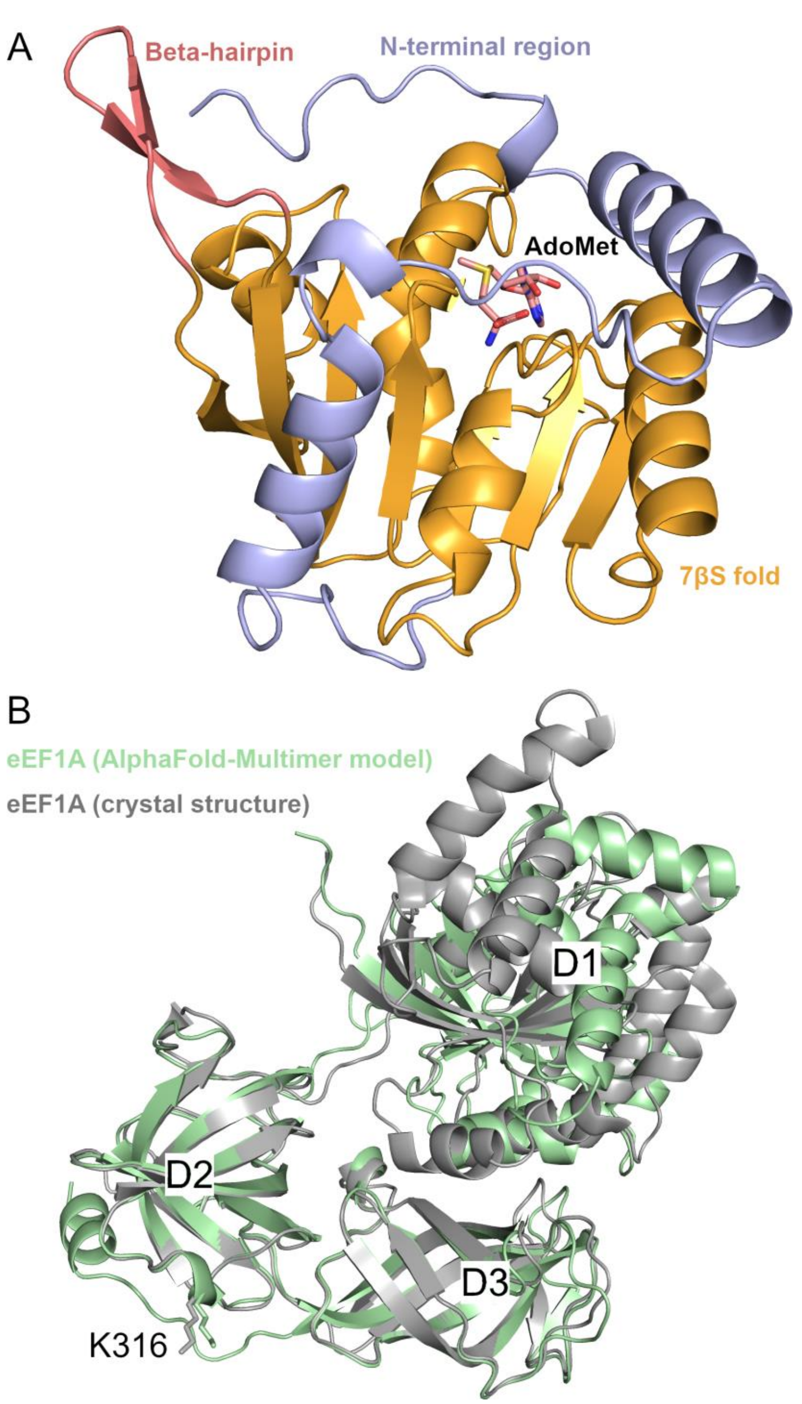

Figure S2. Efm4 and eEF1A from rank 1 AlphaFold-Multimer model show expected folded domains.

A) Efm4 from rank 1 model of Efm4:eEF1A showing the expected 7βS methyltransferase fold (orange), with a short beta-hairpin interrupting the 7βS fold between β6 and β7, as well as an N-terminal region with two additional α-helices. Note that this N-terminal region is different (and after in sequence) from the untranslated upstream region (which is not shown). B) eEF1A from rank 1 model of Efm4:eEF1A aligned with a crystal structure of eEF1A (PDB ID: 1F60). Structures were aligned using domains eEF1A 2 and 3 (RMSD = 0.514 Å). Conformational flexibility of domain 1 (D1) is the only substantial difference between the two structures, with lysine 316 on domain 2 (D2) in an identical position in both structures.

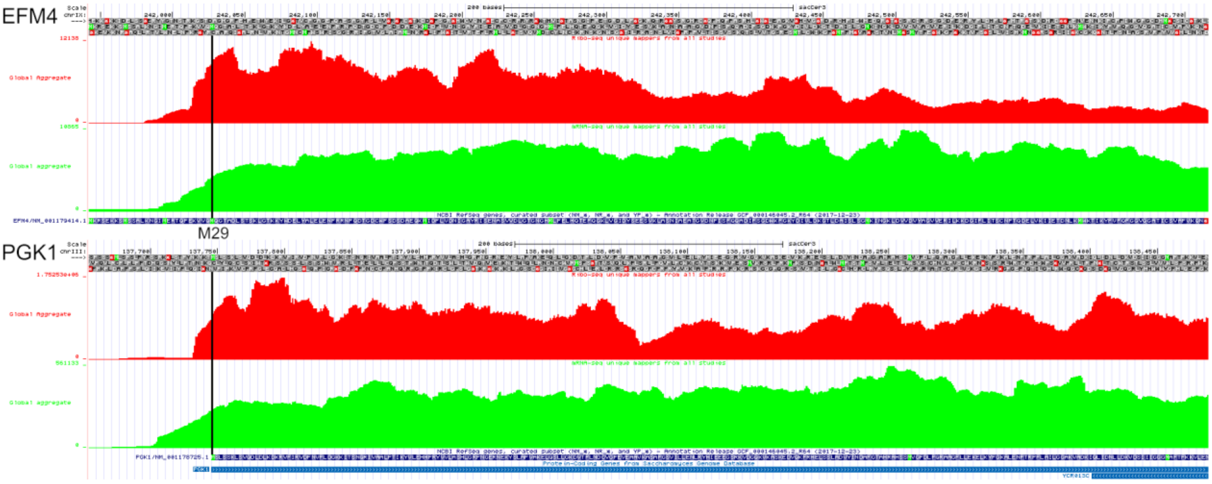

Figure S3. The EFM4 gene begins at the currently annotated M29 position, chrIX:242,027.

Screenshot from the GWIPs-viz browser shows public ribosome footprint profiling (ribo-seq, red) and mRNA-seq (green) data for EFM4 and a reference gene, PGK1. The currently annotated M29 appears to be the true start codon for EFM4. This would place EFM4 at genomic co-ordinates chrIX:242,027 – 242,716 instead of chrIX:241,943 – 242,716.

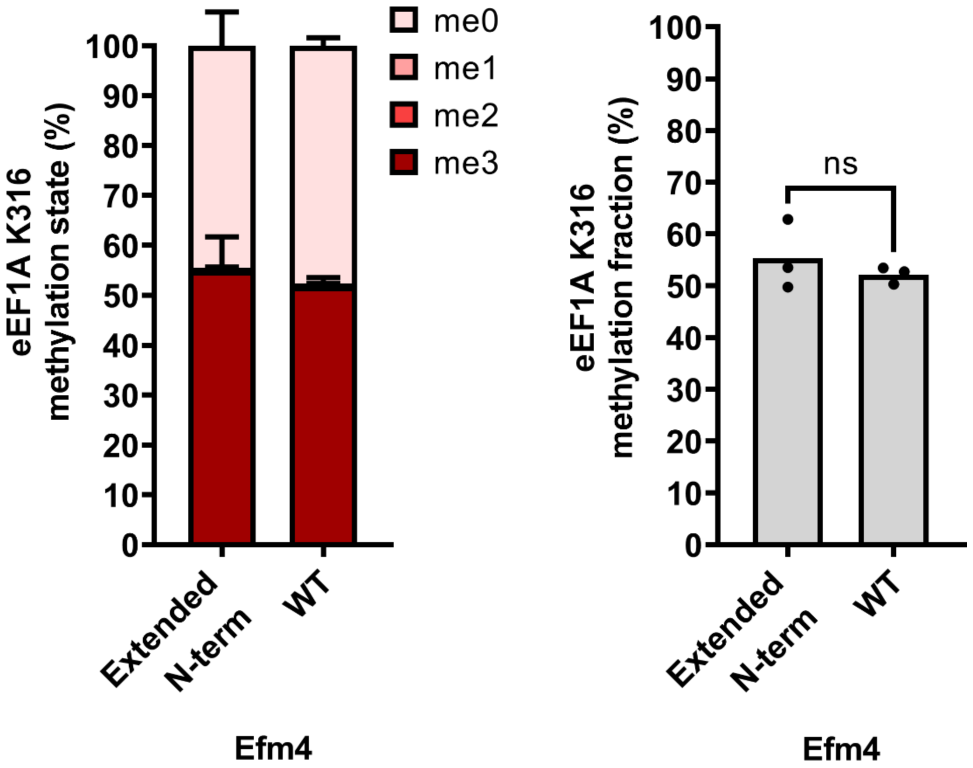

Figure S4. Deletion of incorrectly annotated upstream region of Efm4 does not affect its activity *in vitro*.

Purified "Extended N-term" Efm4 (containing the upstream sequence MKRSEKKSMSSALKNGIMERTQPEKVVQ) or WT Efm4 with this upstream sequence removed (starting MQGTADLSTS…) (both at 3.5 µM) were incubated with eEF1A purified from ΔEFM4 yeast (3 µM) at 30 °C for 3 h. The resulting eEF1A K316 methylation was detected by LC-MS/MS and quantification of tryptic peptide NVSVKEIR (K316 underlined) in its doubly-charged state. Left: Relative levels of eEF1A K316 methylation states. Error bars show one standard deviation. Right: eEF1A K316 methylation fraction relative to 100% trimethylated K316. A two-tailed t-test without equal variance was carried out between the methylation fractions from WT and "Extended N-term" Efm4 (ns: not significant).

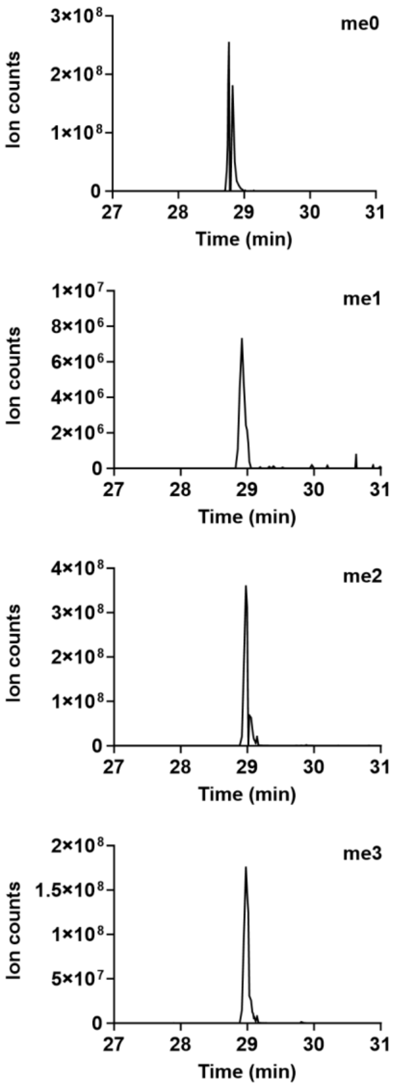

Figure S5. eEF1A K316 methylation generated during the DSSO crosslinking reaction.

Extracted ion chromatograms are shown for the triply-charged form of the eEF1A K316-containing GluC peptide QGVPGDNVGFNVKNVSVKE (K316 underlined) in its un-, mono-, di- and tri-methylated states (me0, me1, me2, me3).

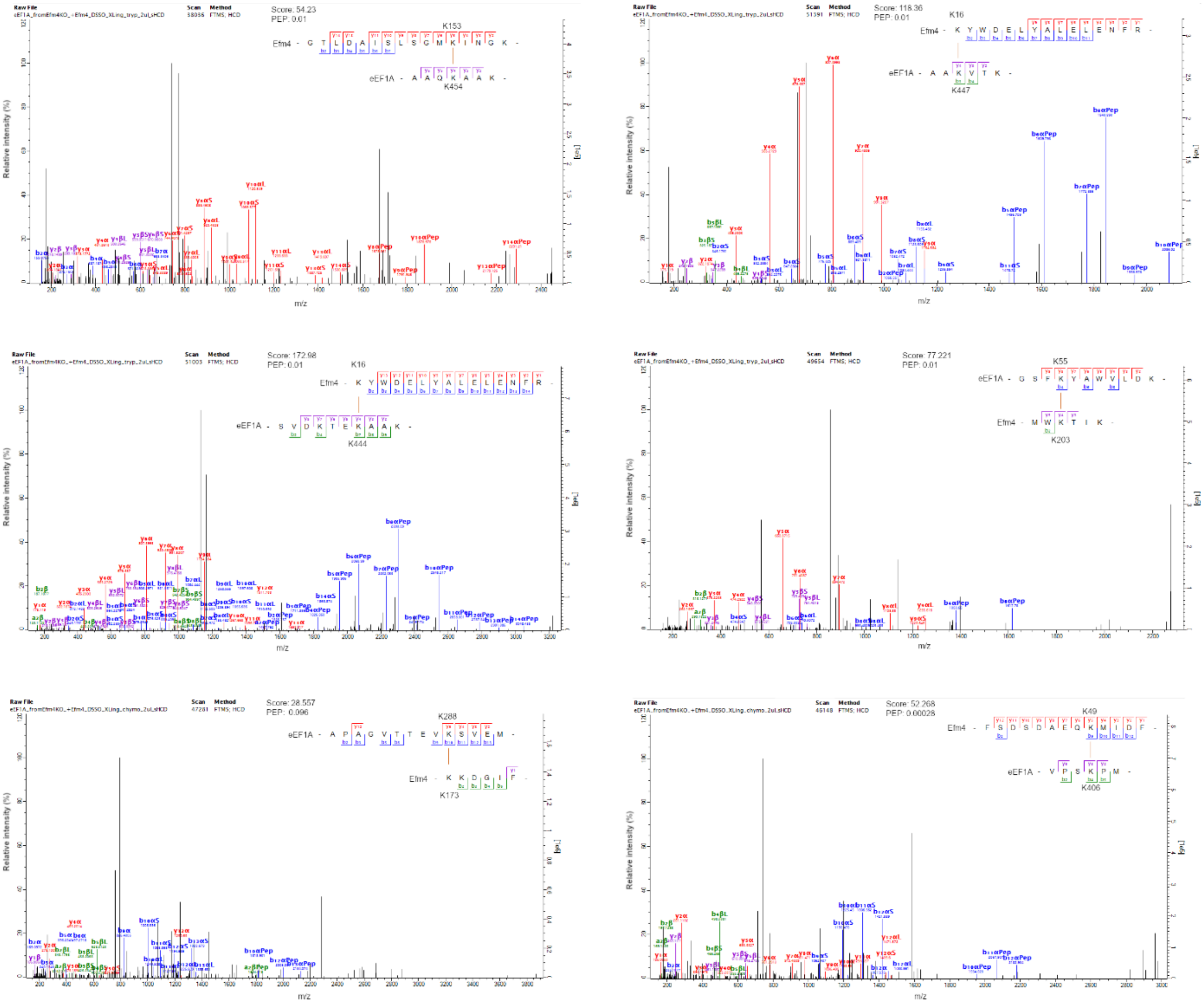

Figure S6. MS/MS spectra identifying DSSO crosslinks between Efm4 and eEF1A.

Purified Efm4 and eEF1A were crosslinked by DSSO in the presence of AdoMet. Proteins were digested with either trypsin, chymotrypsin or GluC, and analysed by LC-MS/MS. Crosslinked peptides were identified using MaxQuant.

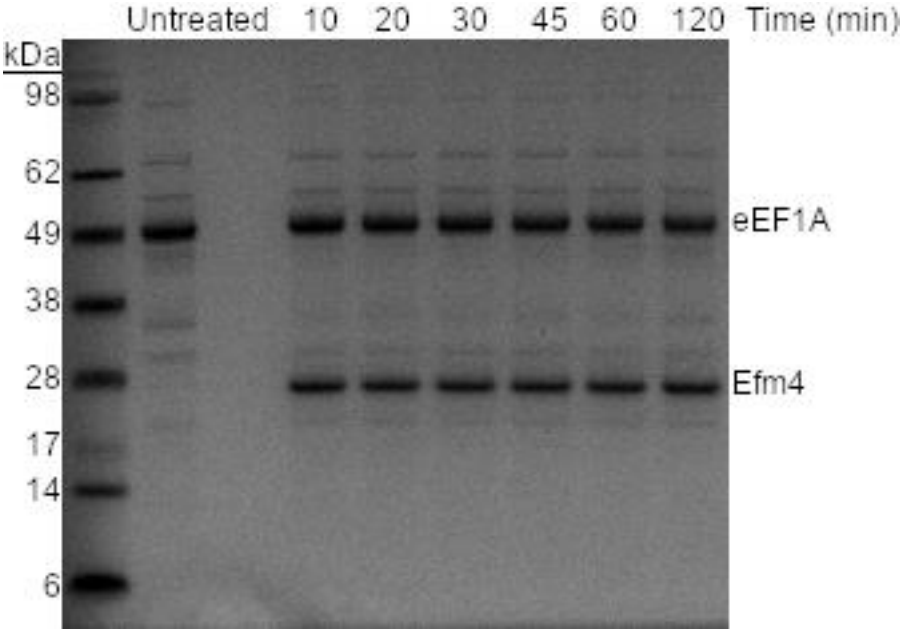

Figure S7. Coomassie-stained SDS-PAGE gel of Efm4 time series assay.

Purified WT Efm4 (3 µM) was incubated with eEF1A (from ΔEFM4) (2 µM) in the presence of AdoMet at 30 °C, for the indicated times. Proteins were then separated by SDS-PAGE and stained with Coomassie. eEF1A gel bands were then digested with AspN and the resulting eEF1A K316 methylation was detected by LC-MS/MS (see Fig. 2A).

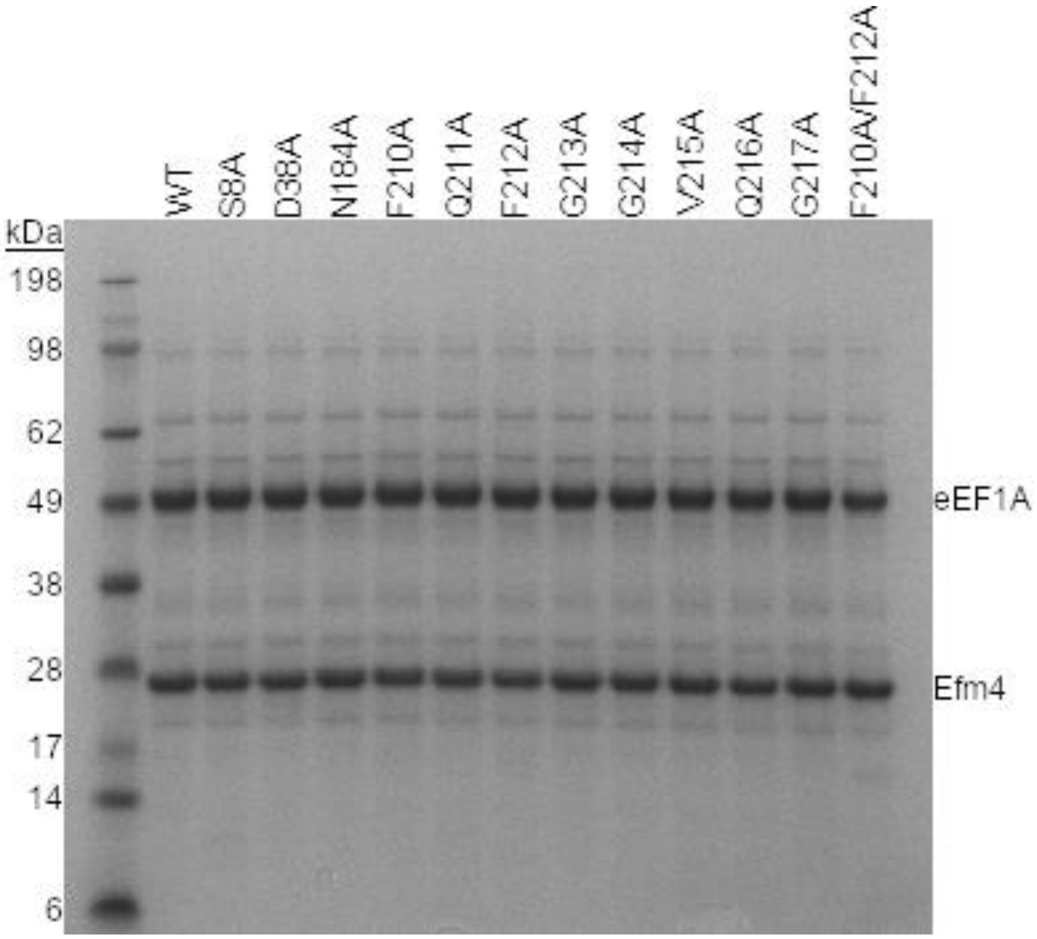

Figure S8. Coomassie-stained SDS-PAGE gel of Efm4 mutant assays.

Purified WT or mutant Efm4 (3 μM) were incubated with eEF1A (from ΔEFM4) (2 μM) in the presence of AdoMet for 30 min at 30 °C. Assays were carried out in triplicate; shown here is a representative replicate. Proteins were separated by SDS-PAGE and stained with Coomassie. eEF1A gel bands were then digested by AspN and the resulting eEF1A K316 methylation was detected by LC-MS/MS (see Fig. 3C).

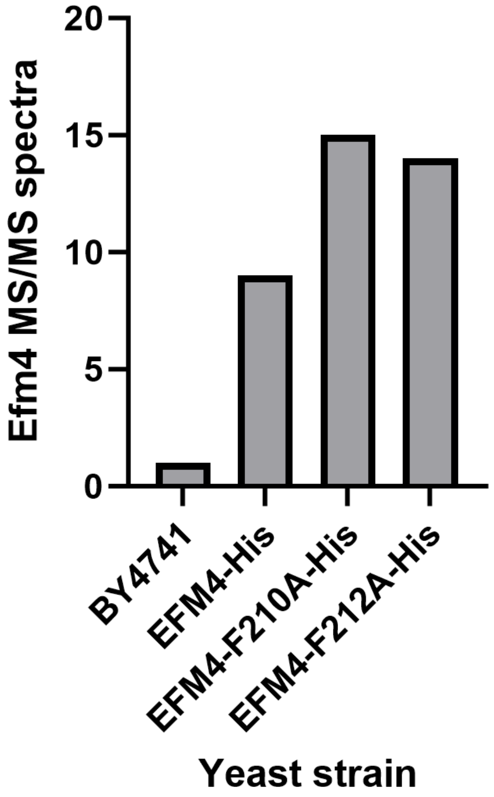

Figure S9. Genomic mutations of EFM4 do not substantially affect its expression.

Efm4 was enriched from strains BY4741, EFM4-His, EFM4-F210A-His and EFM4-F212A-His using His Mag Separose Ni resin. Eluates were separated by SDS-PAGE, the region of the gel corresponding to the size of Efm4 was excised and resulting gel bands were digested with trypsin and analysed by LC-MS/MS. Shown are the resulting number of MS/MS spectra matching to Efm4.

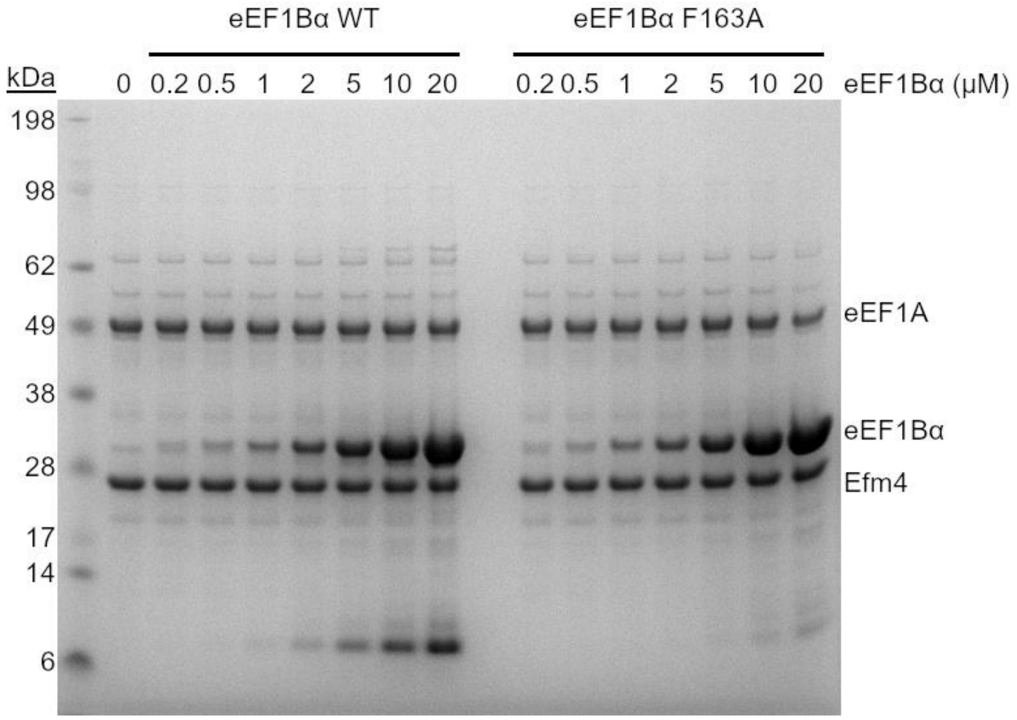

Figure S10. Coomassie-stained SDS-PAGE gel of Efm4 assays with WT and F163A eEF1Bα.

Purified WT Efm4 (3 μM) was incubated with eEF1A (from ΔEFM4) (2 μM) in the presence of varying concentrations of either wild-type or F163A eEF1Bα and with AdoMet for 30 min at 30 °C. Assays were carried out in triplicate; shown here is a representative replicate. Proteins were separated by SDS-PAGE and stained with Coomassie. eEF1A gel bands were then digested by AspN and the resulting eEF1A K316 methylation was detected by LC-MS/MS (see Fig. 4B).

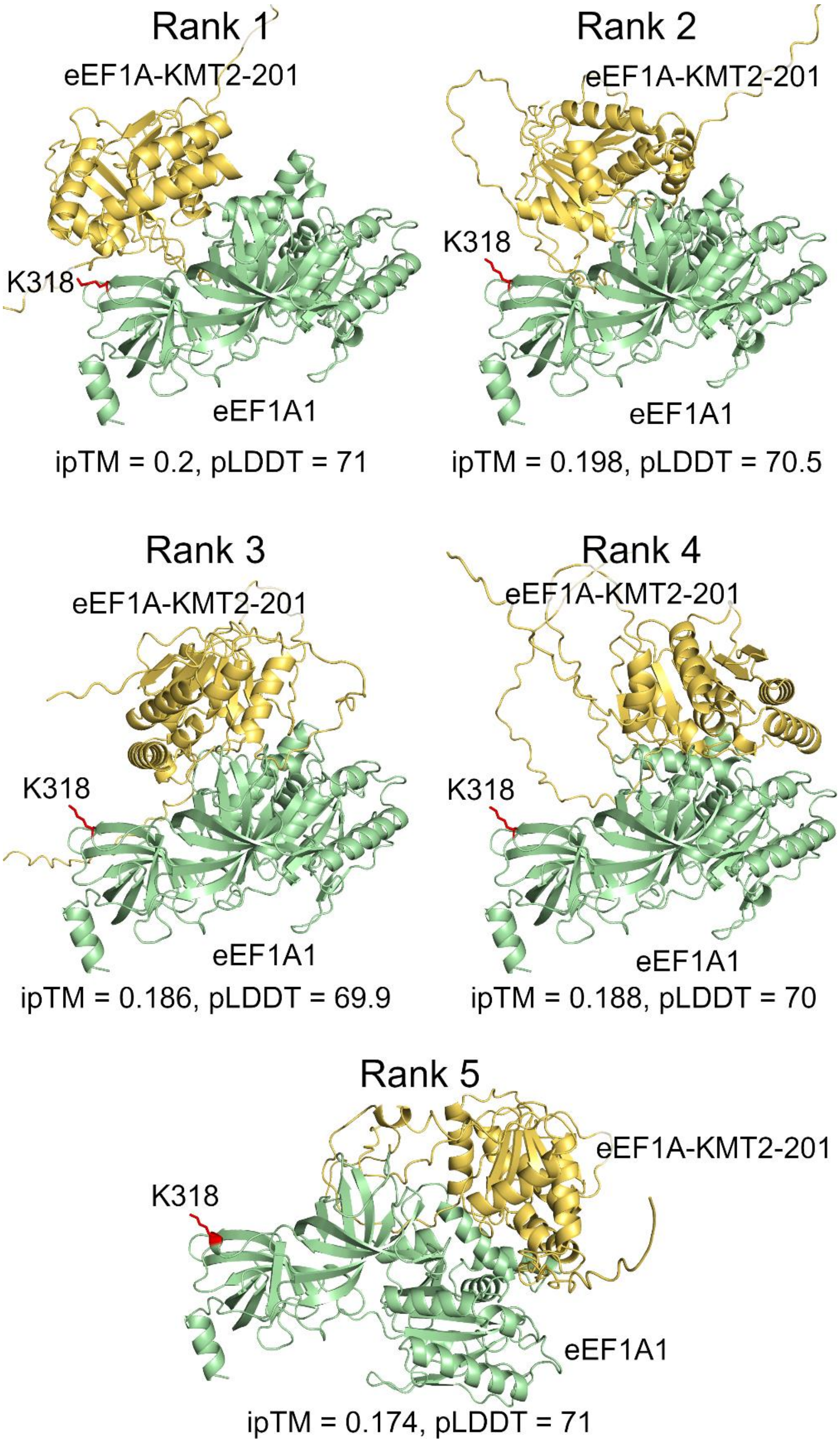

Figure S11. AlphaFold-Multimer models of eEF1A-KMT-201 bound to eEF1A1.

AlphaFold-Multimer models of eEF1A-KMT2-201 bound to eEF1A1 were generated as described in methods. eEF1A1 K318 is not bound at the active site of eEF1A-KMT2, as would be expected.

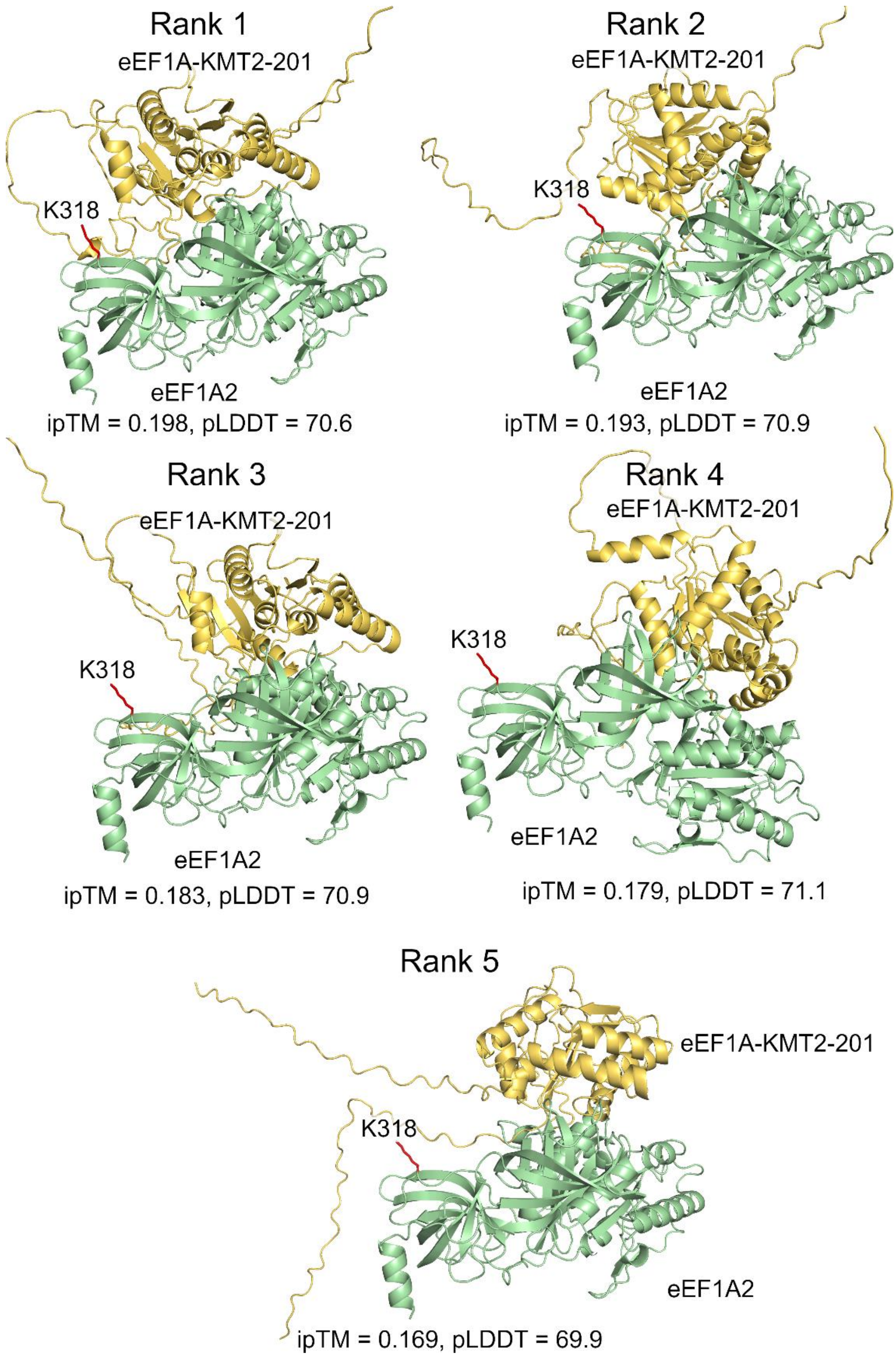

Figure S12. AlphaFold-Multimer models of eEF1A-KMT-201 bound to eEF1A2.

AlphaFold-Multimer models of eEF1A-KMT2-201 bound to eEF1A2 were generated as described in methods. eEF1A2 K318 is not bound at the active site of eEF1A-KMT2, as would be expected.

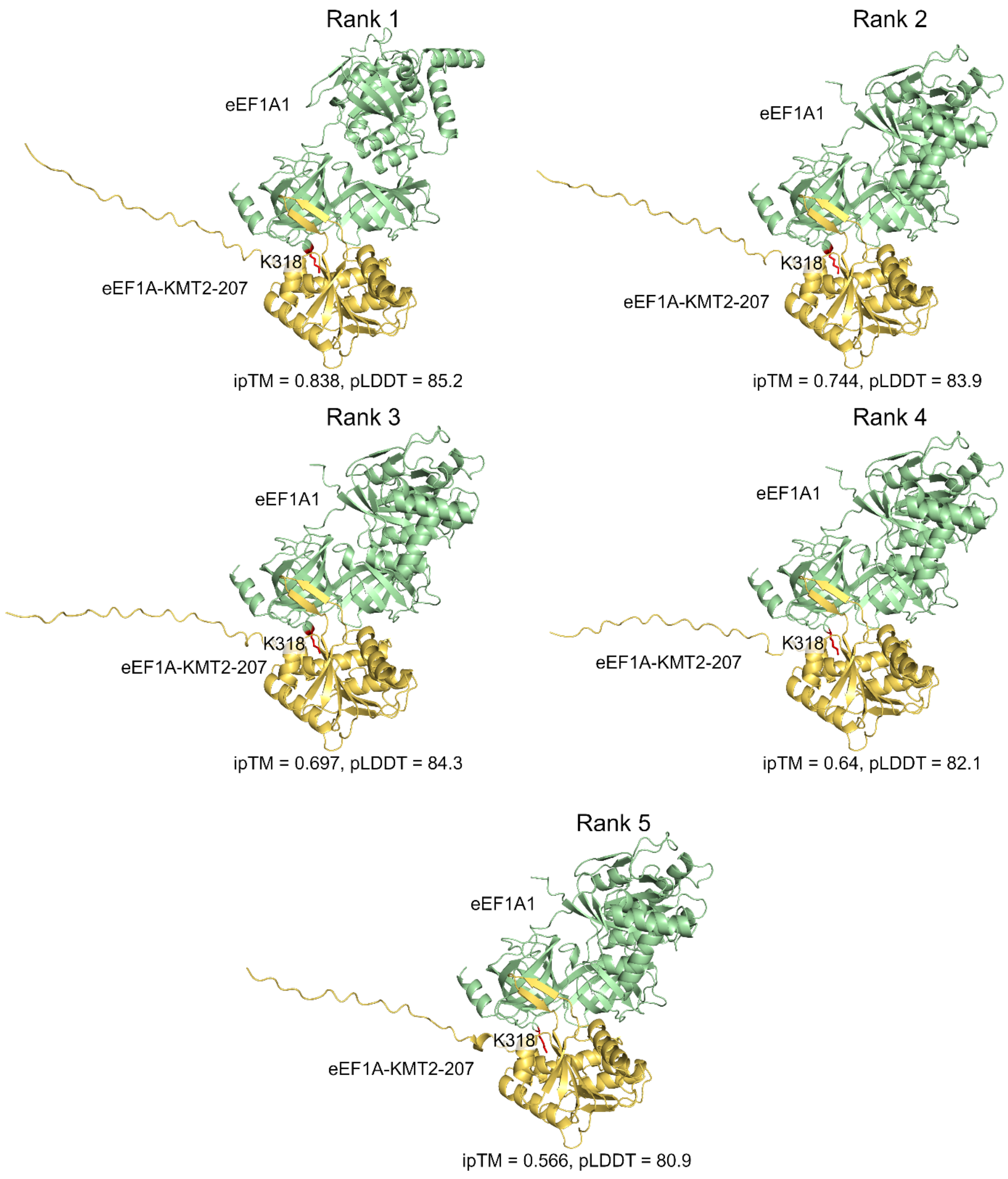

Figure S13. AlphaFold-Multimer models of eEF1A-KMT-207 bound to eEF1A1.

AlphaFold-Multimer models of eEF1A-KMT2-207 bound to eEF1A1 were generated as described in methods. eEF1A1 K318 is bound at the active site of eEF1A-KMT2. The N-terminal 26 residues of eEF1A-KMT2-207 are likely to be unstructured, with pLDDT scores <50.

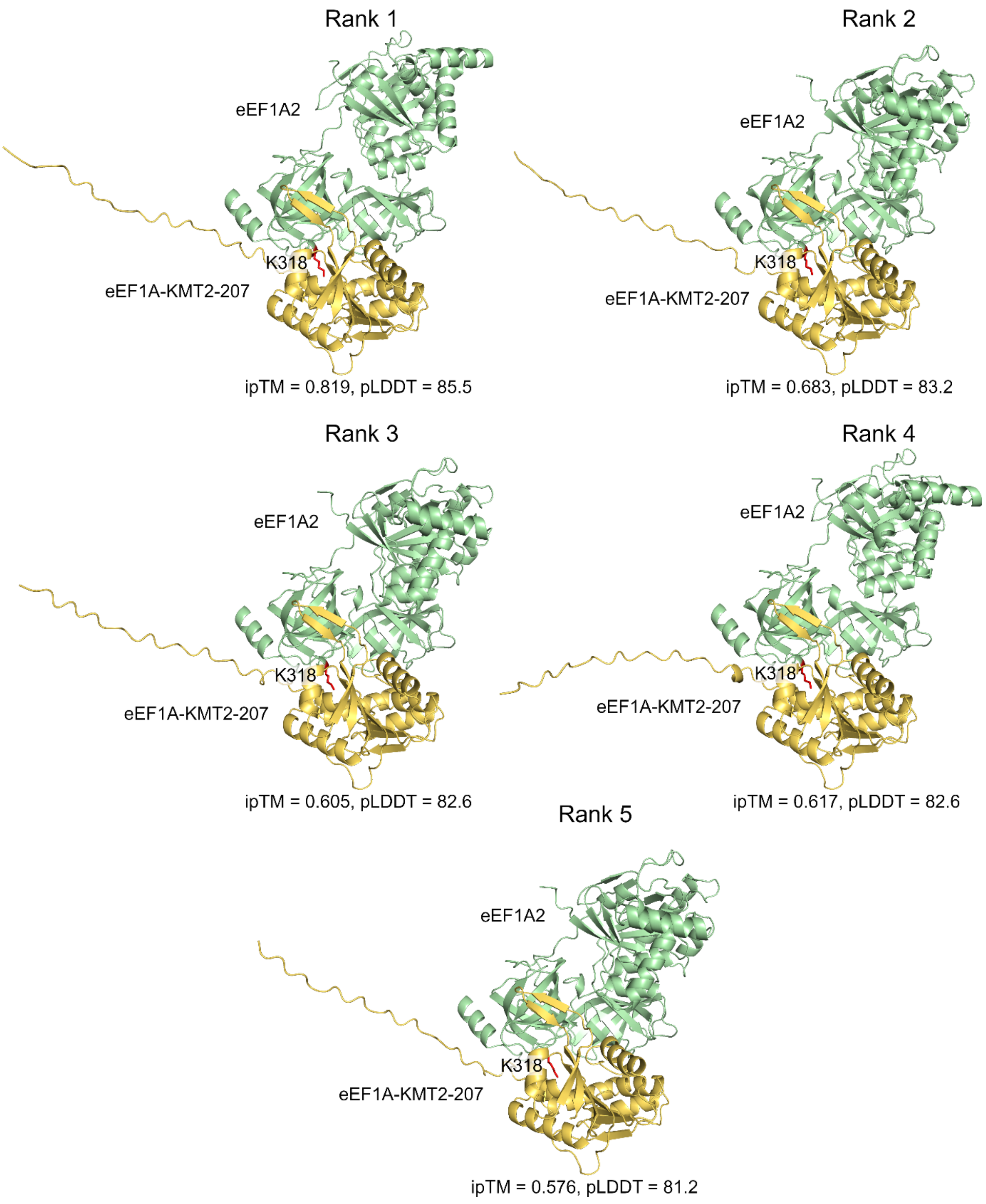

Figure S14. AlphaFold-Multimer models of eEF1A-KMT-207 bound to eEF1A2.

AlphaFold-Multimer models of eEF1A-KMT2-207 bound to eEF1A2 were generated as described in methods. eEF1A2 K318 is bound at the active site of eEF1A-KMT2. The N-terminal 26 residues of eEF1A-KMT2-207 are likely to be unstructured, with pLDDT scores <50.

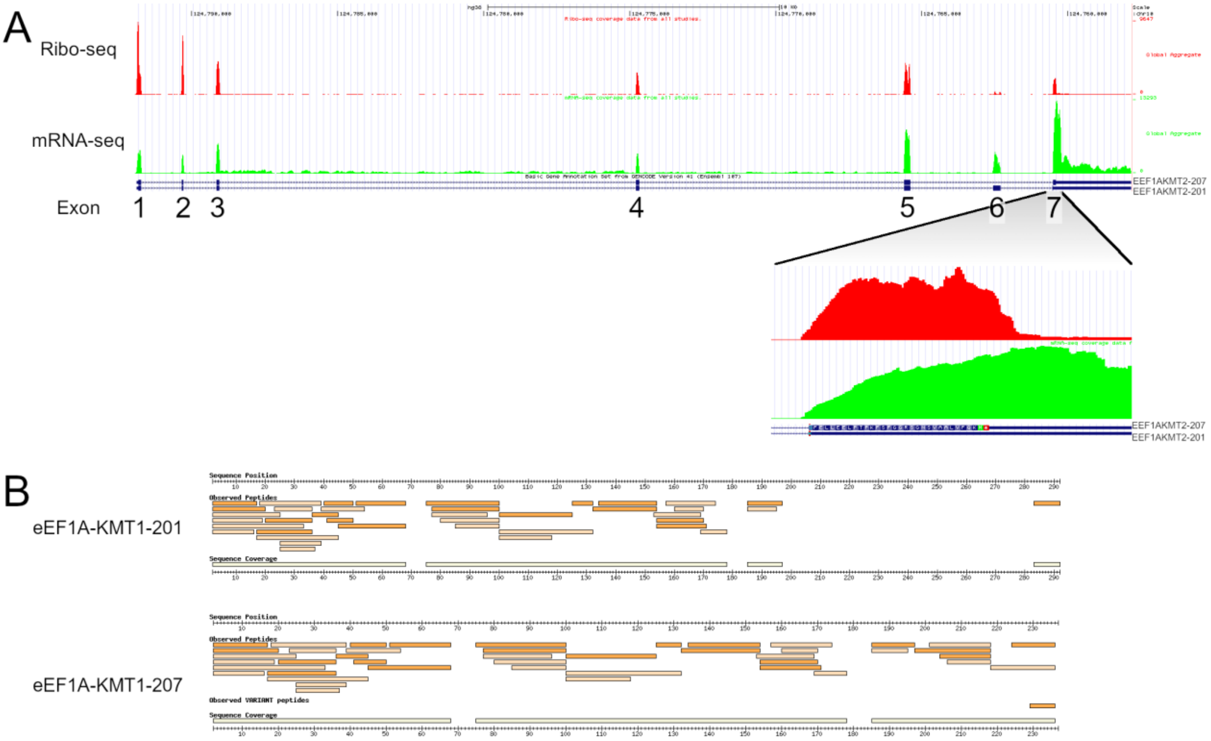

Figure S15. Public mRNA-seq, Ribo-seq and proteomic data indicate that eEF1A-KMT2-207 is the predominantly expressed isoform.

A) Aggregate mRNA-seq and Ribo-seq data for eEF1A-KMT2 isoforms from GWIPS-viz Genome Browser. Exon 6 is substantially less translated than all other exons, including exon 7, indicating that eEF1A-KMT2-207 is translated more than eEF1A-KMT2-201. B) Proteomic evidence for eEF1A-KMT2-201 and eEF1A1-KMT2-207 from PeptideAtlas, showing higher sequence coverage for eEF1A-KMT2-207.

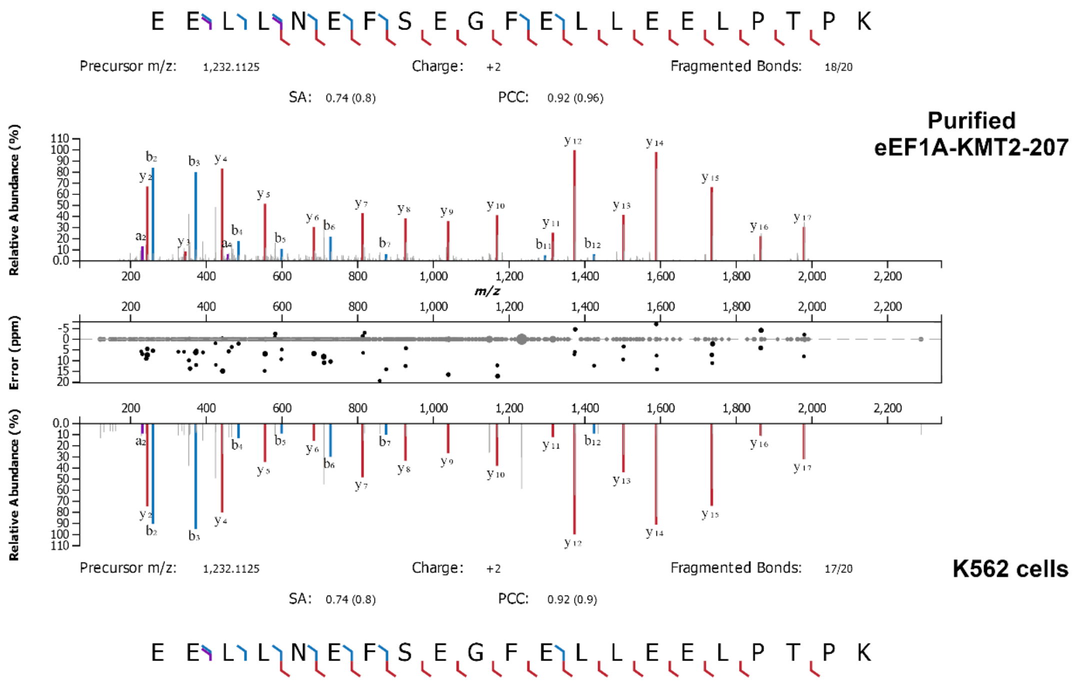

USI: mzspec:PXD005141:20151123_QEp1_LC7_NiKu_SA_K562_Trypsin-bRP02:scan:88296:EELLNEFSEGFELLEELPTPK/2

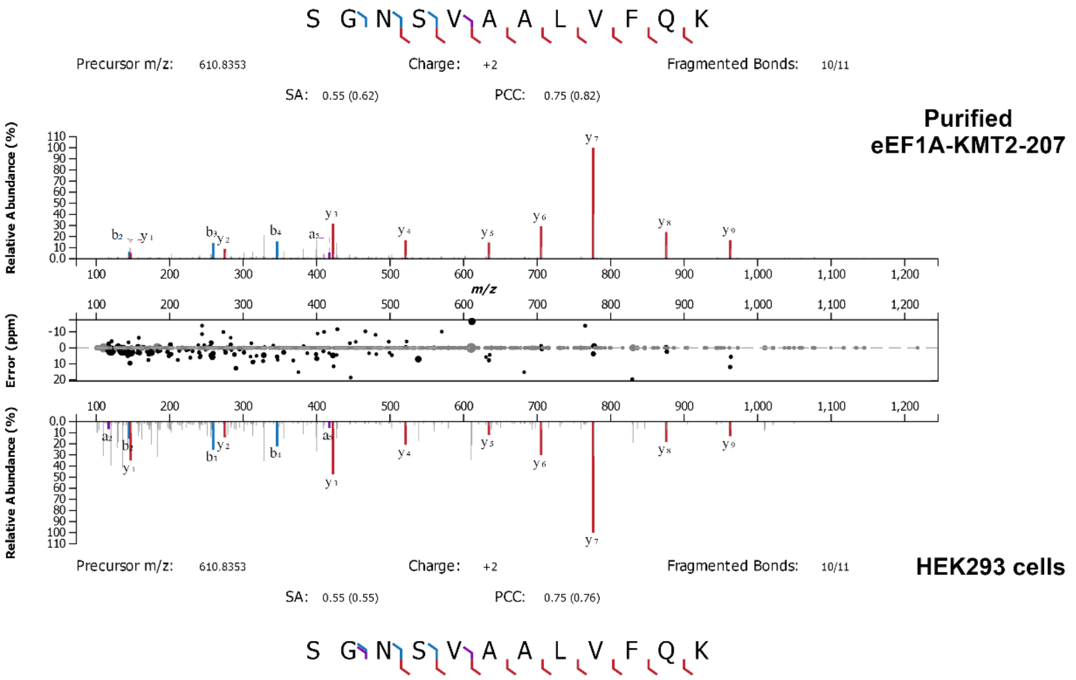

USI: mzspec:PXD005141:20151123_QEp1_LC7_NiKu_SA_Hek293_Trypsin-bRP07:scan:51314:SGNSVAALVFQK/2

Figure S16. Comparison of MS/MS spectra confirm identity of eEF1A-KMT2-207-specific peptides in cell lines.

Comparison of MS/MS spectra generated from purified eEF1A-KMT2-207 and from public proteomic datasets, confirming the identity of two eEF1A-KMT2-207-specific peptides. Spectra were graphed and compared using the Universal Spectrum Explorer, with the following settings: fragment ions: a, b, y, fragment ion charge states: 1+, 2+; fragment annotation tolerance: 20 ppm; annotation intensity threshold: 5% base peak.

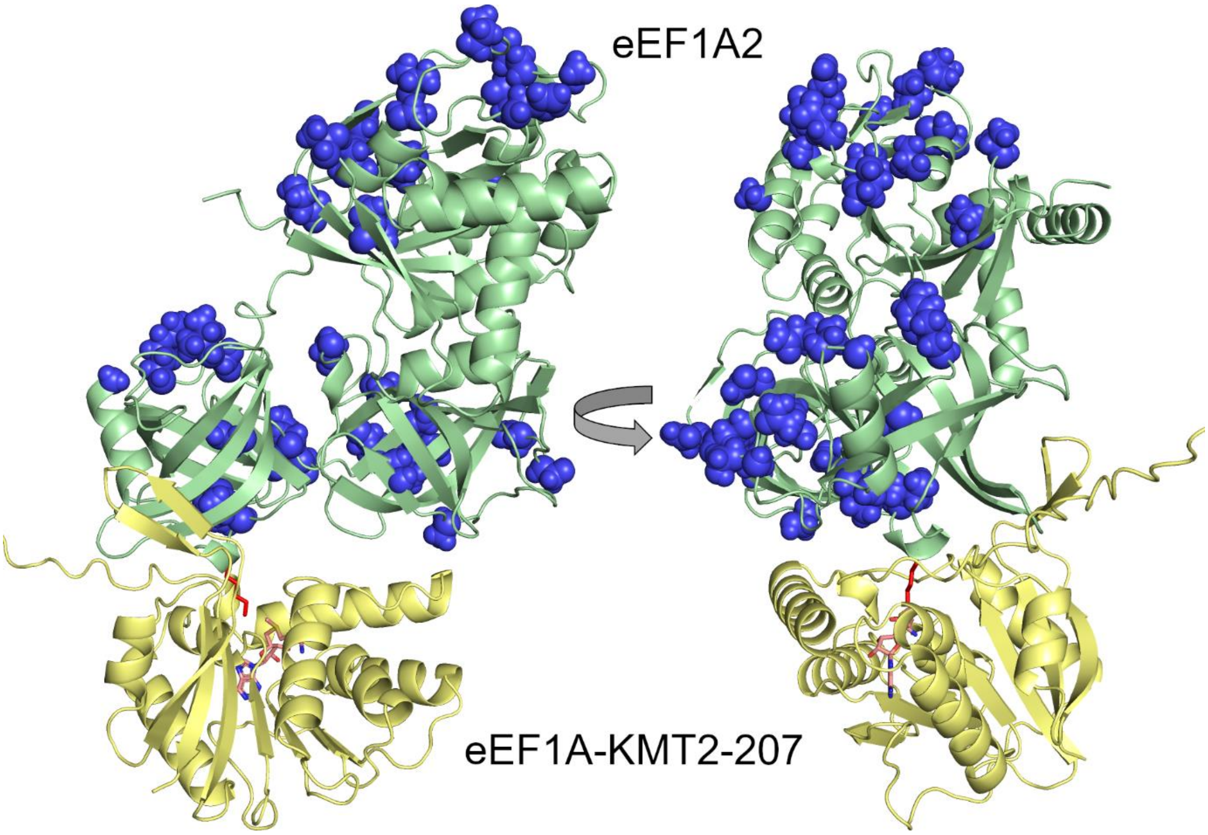

Figure S17. Almost all residues unique to eEF1A2 are not at the interface with eEF1A-KMT2.

Top-ranked AlphaFold-Multimer model of eEF1A-KMT2-207:eEF1A2 complex (shown as cartoon), with the residues differing between eEF1A2 and eEF1A1 shown as blue spheres.

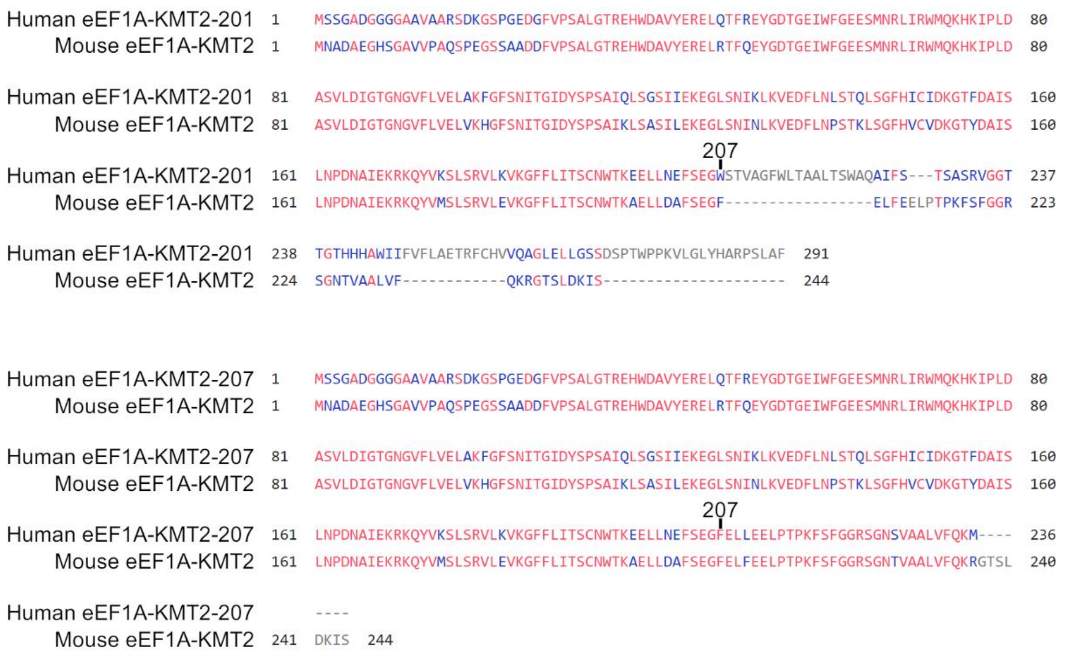

Figure S18. Mouse eEF1A-KMT2 is more similar to human eEF1A-KMT2-207 than eEF1A-KMT2-201.

Alignments of mouse eEF1A-KMT2 (UniProt ID Q9D853) with human eEF1A-KMT2-201 (UniProt ID Q5JPI9) or human eEF1A-KMT2-207 (UniProt ID A0A494BZY7) were generated using COBALT (NBCI). Identical residues are red, different residues are blue and gaps are grey. Residue 207, after which eEF1A-KMT2-201 and eEF1A-KMT2-207 diverge in sequence, is indicated.

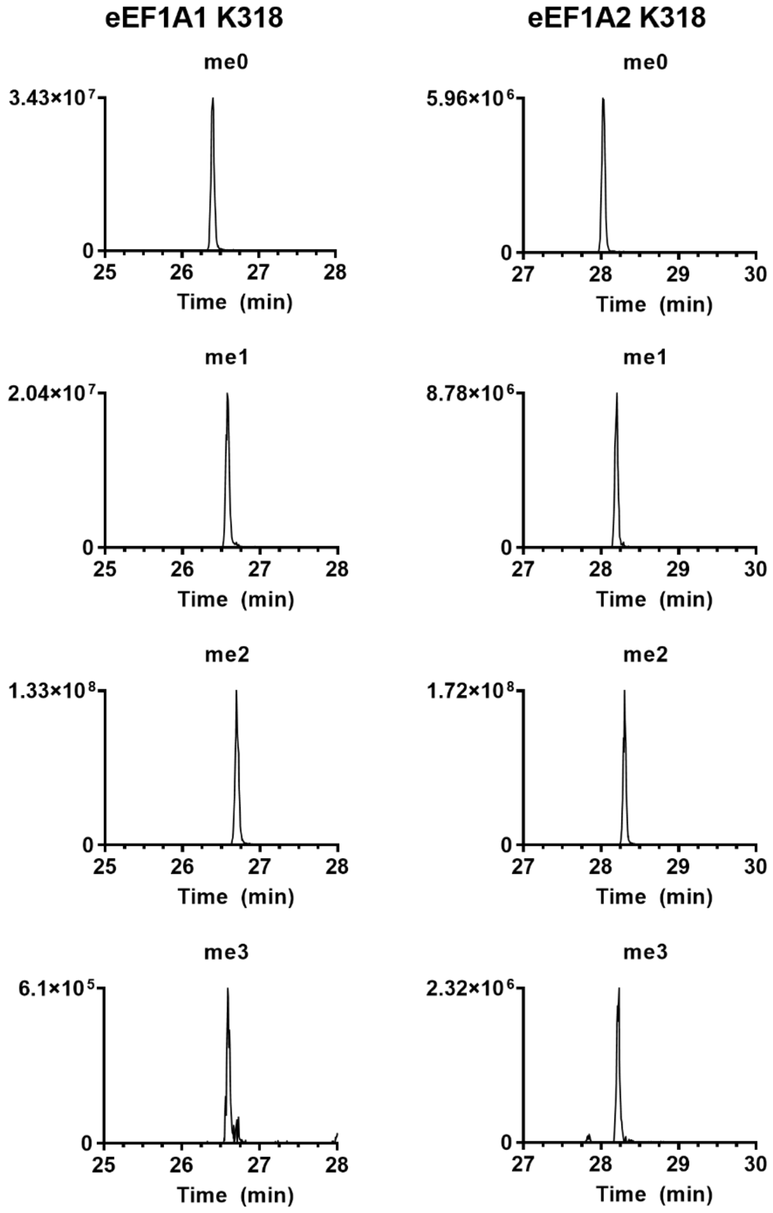

Figure S19. Human eEF1A1 and eEF1A2 purified from WT yeast are methylated at K318.

Data from Hamey et al. 2017 (PRIDE: PXD005497) were interrogated the presence of eEF1A K318 methylation. Specifically, raw files "Invitro_Methylation_assay_eEF1A1_negative_trypsin.raw" (eEF1A1) or "Invitro_Methylation_assay_eEF1A2_negative_trypsin.raw" (eEF1A2) were used. The K318 methylation status of eEF1A1 and eEF1A2 were measured by taking extracted ion chromatograms (XICs) of doubly-charged tryptic peptides NVSVKDVR (eEF1A1) or NVSVKDIR (eEF1A2) in all methylation states.

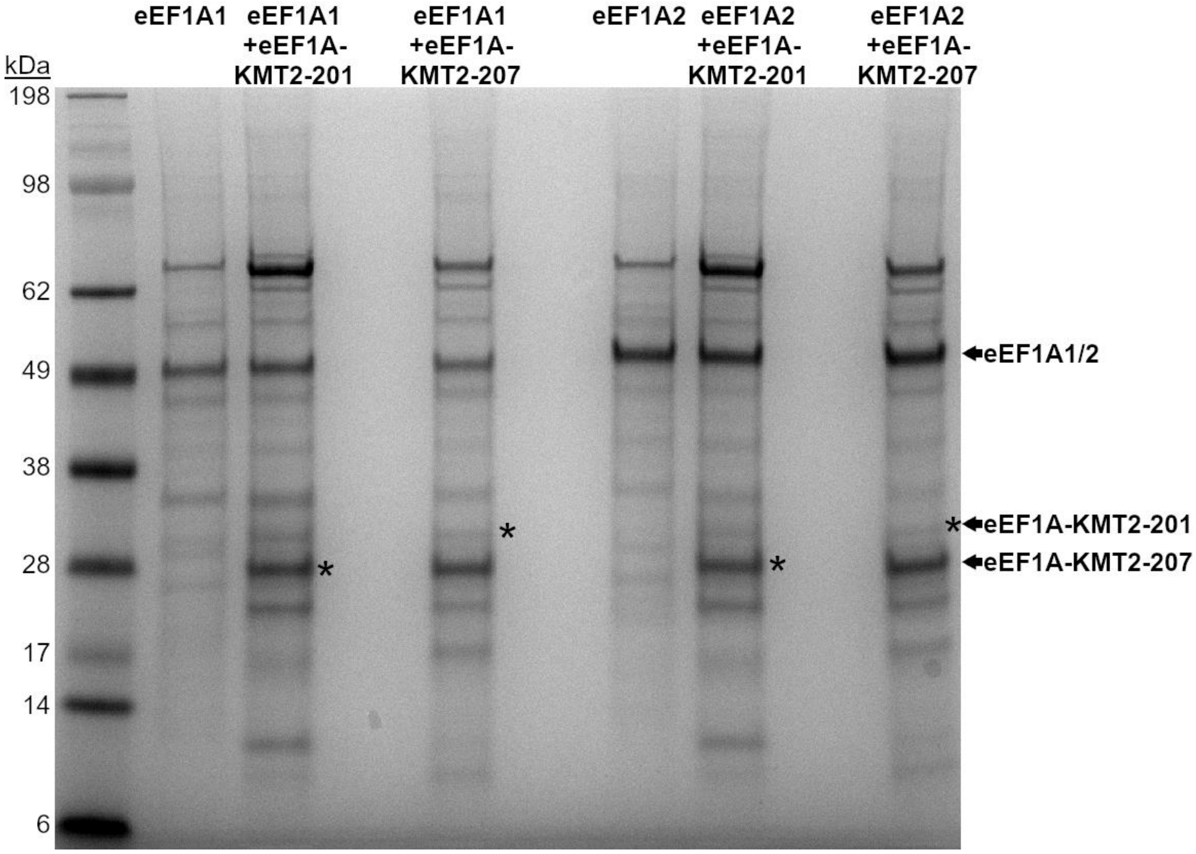

Figure S20. Coomassie-stained SDS-PAGE gel of eEF1A-KMT2-201 and eEF1A-KMT2-207 methylation assays of eEF1A1 and eEF1A2.

Purified eEF1A1 or eEF1A2 (2.2 μM) were incubated without any enzyme or with eEF1A-KMT2-201 or eEF1A-KMT2-207 (3 μM) in the presence of AdoMet for 18 h at 37 °C. Proteins were separated by SDS-PAGE and stained with Coomassie. eEF1A1 and eEF1A2 gel bands were then digested by AspN and analysed by LC-MS/MS (see Fig. 6B). For the eEF1A1 assay, bands corresponding to eEF1A-KMT2-201 and eEF1A-KMT2-207 were also excised, digested with trypsin and the presence of the enzymes verified by LC-MS/MS. eEF1A-KMT2-201 and eEF1A-KMT2-207 were high abundance in the analysed bands, being the top hit besides eEF1A in both cases. Asterisk (*) indicates contaminating proteins from *E. coli*.

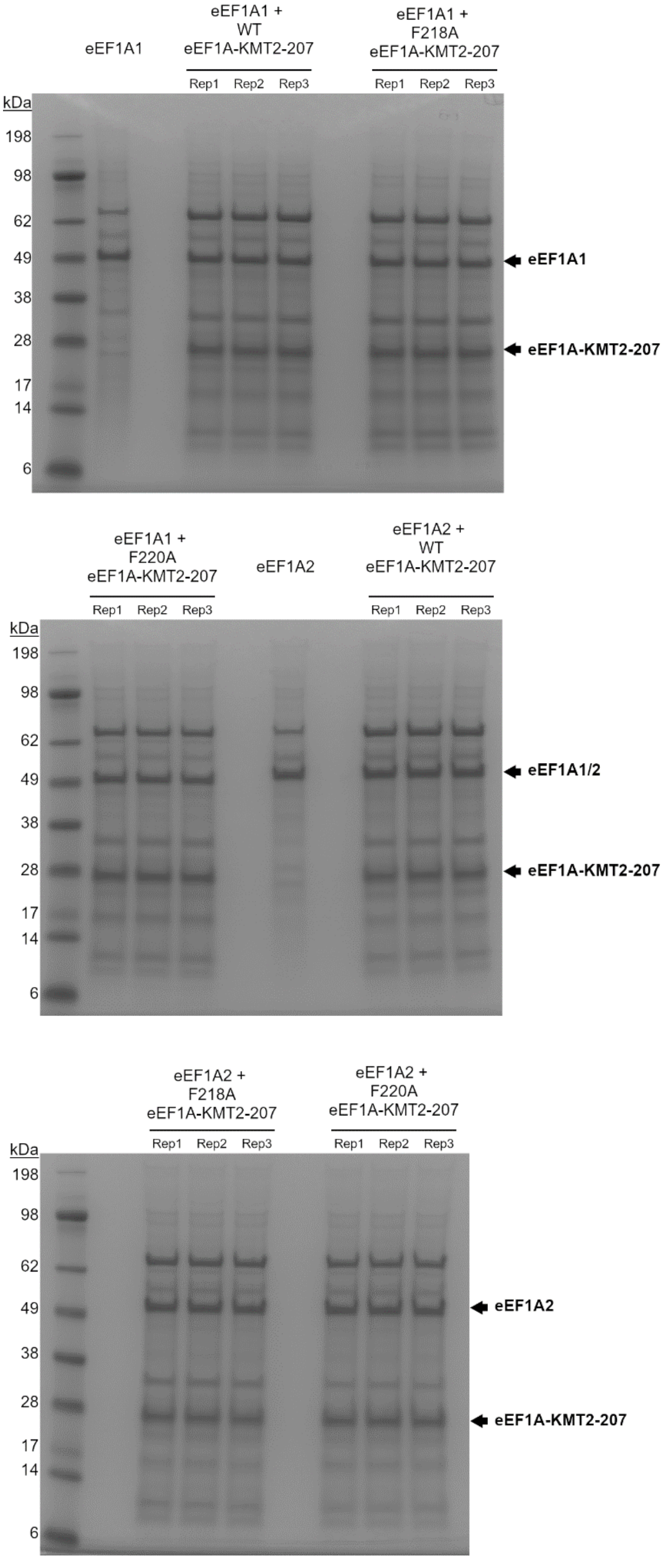

Figure S21. Coomassie-stained SDS-PAGE gels of eEF1A-KMT2-207 F218A and F220A mutant assays.

Purified WT and mutant eEF1A-KMT-207 (3 μM) were incubated with eEF1A1 or eEF1A2 (both 2.2 μM) in the presence of AdoMet for 2 h at 37 °C. Proteins were separated by SDS-PAGE and stained with Coomassie. eEF1A1/2 gel bands were then digested with AspN and the resulting eEF1A1/2 K318 methylation was detected by LC-MS/MS (see Fig. 7C, D).

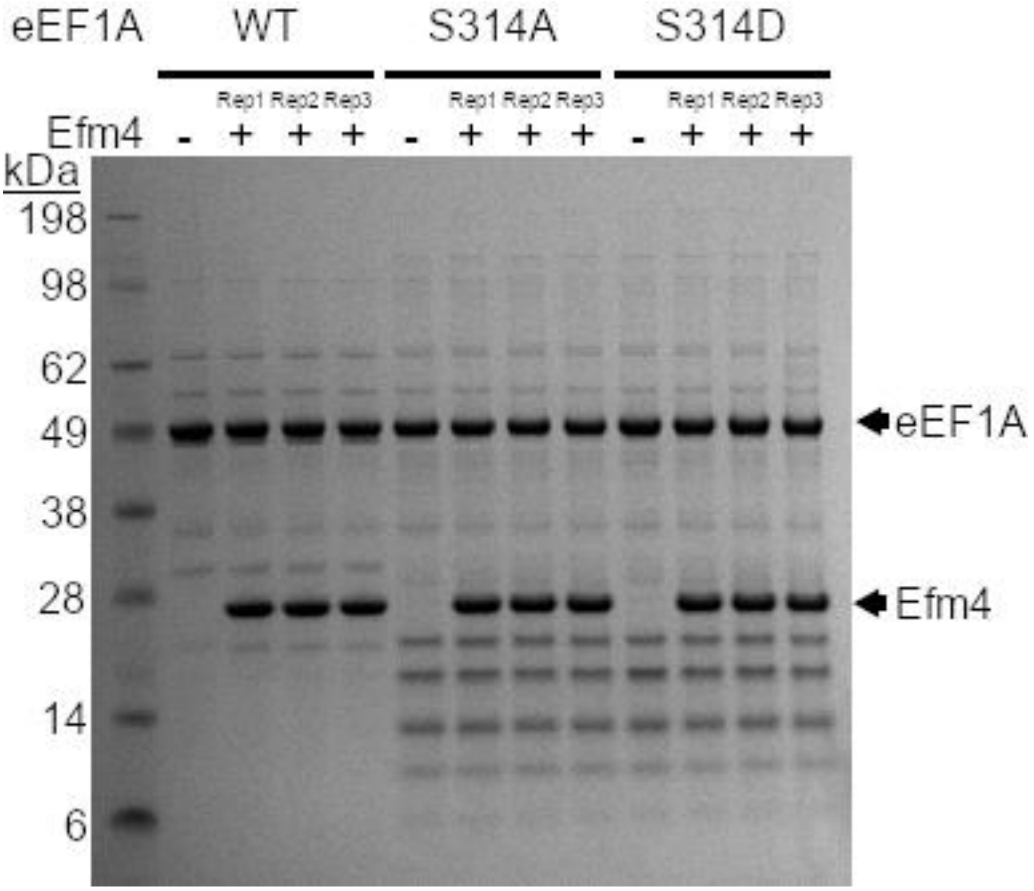

Figure S22. Coomassie-stained SDS-PAGE gels of eEF1A phospho-mutant assays.

Purified WT and mutant eEF1A (from ΔEFM4) (2 μM) were incubated with or without purified Efm4 (3 μM) in the presence of AdoMet at 30 °C for 30 min. Proteins were separated by SDS-PAGE and stained with Coomassie. eEF1A gel bands were then digested with trypsin and the resulting K316 methylation was detected by LC-MS/MS (see Fig. 8C).

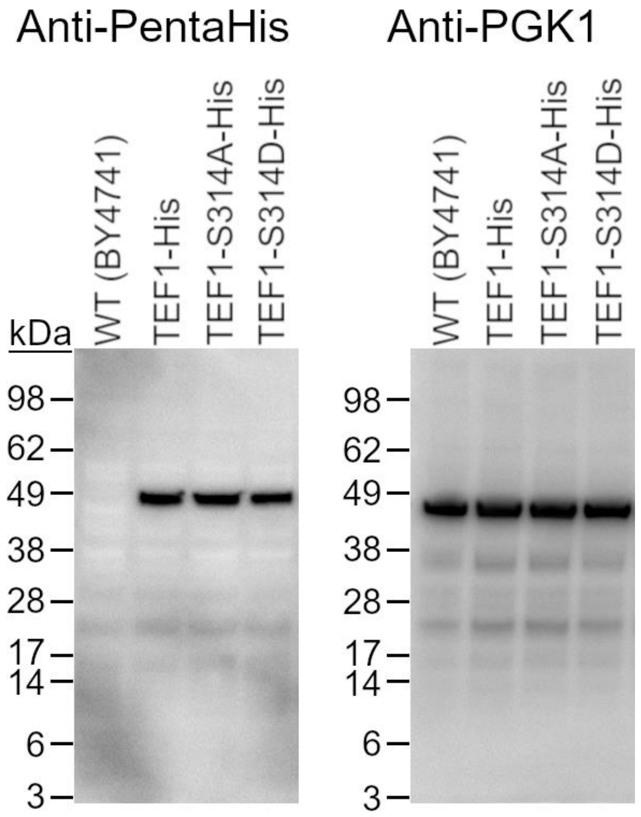

Figure S23. PentaHis immunoblot confirms phospho-mutants have no effect on eEF1A protein levels.

Levels of hexahistidine-tagged WT and S314-mutant eEF1A were detected in lysates from WT (BY4741), TEF1-His, TEF1-S314A-His and TEF1-S314D-His yeast strains via immunoblotting with an anti-Penta-His HRP antibody. The WT strain showed no signal, as expected, while WT hexahistidine-tagged eEF1A (from TEF1-His strain) showed similar levels to hexahistidine-tagged and S314A or S314D mutant eEF1A (from TEF1-S314A-His and TEF1-S314D-His strains). Lysates were also immunoblotted with an anti-PGK1 antibody as a loading control.

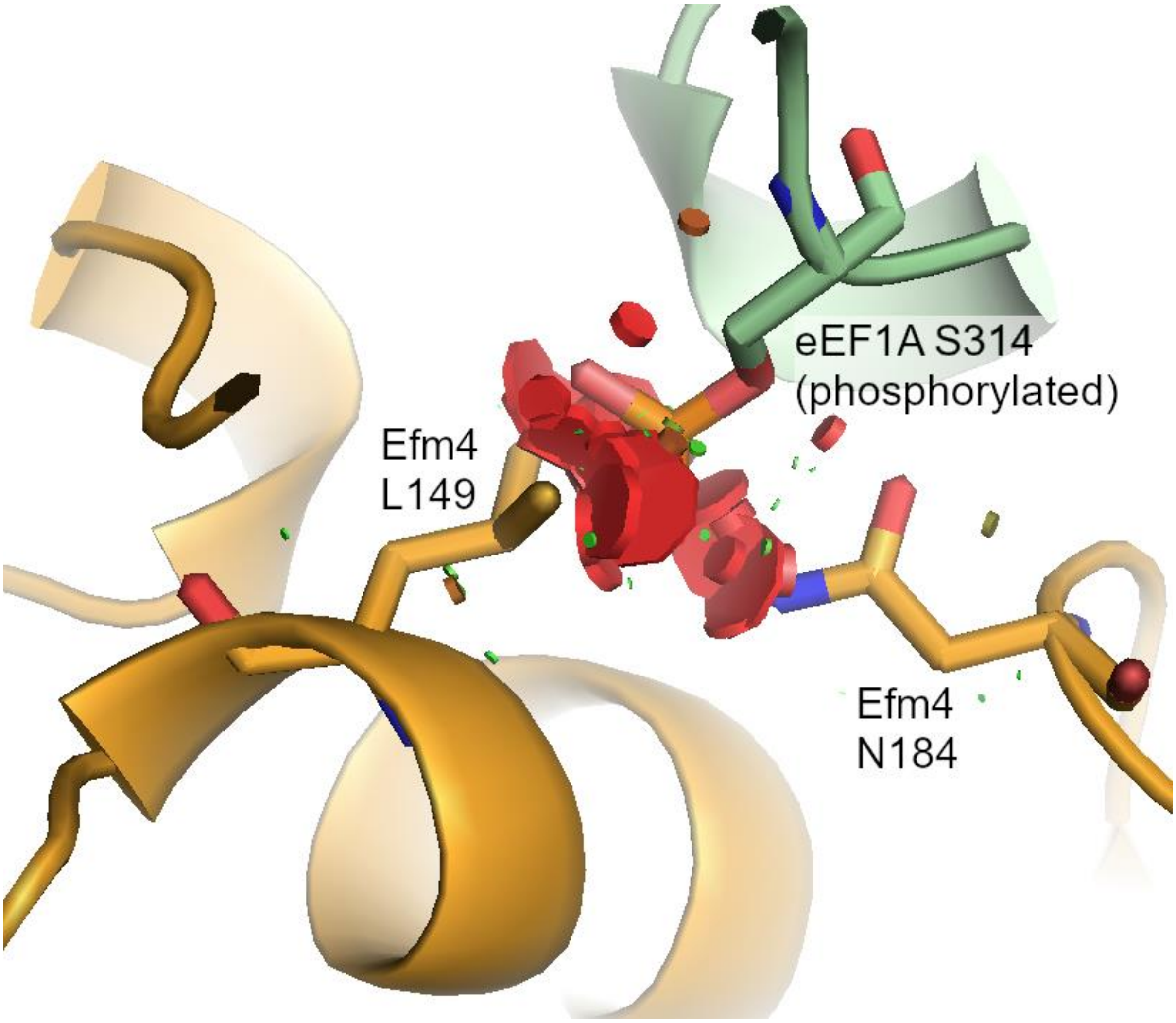

Figure S24. Phosphorylated eEF1A S314 likely causes steric clashes with Efm4 L149 and N184.

eEF1A S314 in the AlphaFold-Multimer model of Efm4:eEF1A (rank 1) was changed to a phospho-serine, and the resulting steric clashes with Efm4 are shown as red discs.
